# BIRC5 upregulation enhances DNMT3A-mutant T-ALL cell survival and pathogenesis

**DOI:** 10.1016/j.bneo.2024.100040

**Published:** 2024-09-05

**Authors:** Wangisa Dunuwille, William C. Wilson, Hassan Bjeije, Nancy Issa, Wentao Han, Tyler M. Parsons, Andrew L. Young, Infencia Xavier Raj, Aishwarya Krishnan, Tarang Gaur, Eunice S. Wang, Andrew P. Weng, Matthew C. Stubbs, Hamza Celik, Amanda F. Cashen, John R. Edwards, Grant A. Challen

**Affiliations:** 1 Division of Oncology, Department of Medicine, Washington University School of Medicine, St. Louis, MO; 2 Division of Hematology, Department of Medicine, Washington University School of Medicine, St. Louis, MO; 3 Department of Immunology, Roswell Park Comprehensive Cancer Center, Buffalo, NY; 4 Department of Pathology and Laboratory Medicine, Terry Fox Laboratory, BC Cancer Agency, Vancouver, BC, Canada; 5 Myeloid Biology Group, Incyte Research Institute, Wilmington, DE; 6 Department of Medicine, Center for Pharmacogenomics, Washington University School of Medicine, St. Louis, MO

## Abstract

T-cell acute lymphoblastic leukemia (T-ALL) is an aggressive hematopoietic neoplasm. Although the prognosis of pediatric T-ALL has improved with intensified chemotherapy regimens, this benefit has largely not translated to the adult T-ALL population. Development of new treatments requires understanding the mechanisms driven by specific mutations. *DNMT3A* mutations are identified in ∼10% to 18% of adult patients with T-ALL and are associated with poor clinical outcomes. Here, using primary human specimens, we show that cells from patients with T-ALL with *DNMT3A* mutations are resistant to apoptosis and certain chemotherapies. Elevated JAK/STAT signaling drove prosurvival programs in patients with mutant *DNMT3A*, and JAK/STAT inhibition restored sensitivity to chemotherapy. The prosurvival gene *BIRC5* was upregulated in patients with *DNMT3A*-mutant T-ALL, and these cells were specifically sensitive to the Baculoviral IAP Repeat Containing 5 (BIRC5) inhibitor YM155. Genetic inhibition of *BIRC5* in vivo lead to rapid depletion of *DNMT3A*-mutant T-ALL cells in patient-derived xenografts, positioning BIRC5 as a precision medicine target for these patients.

## Key Points

• DNMT3A mutations confer enhanced survival to T-ALL cells through increased JAK/STAT signaling.
• BIRC5 represents a specific genetic dependency and therapeutic vulnerability of T-ALL cells with DNMT3A mutations.

## Visual Abstract

**Figure:**
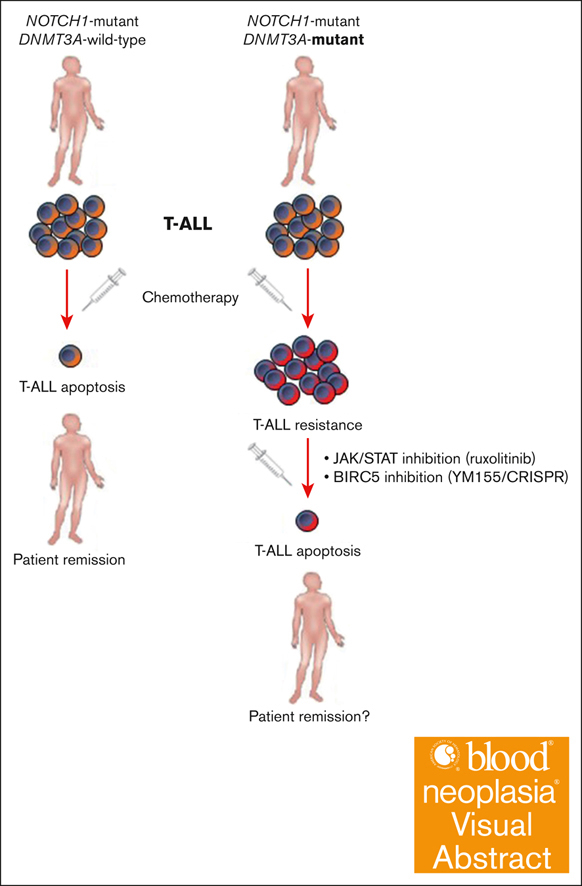

## Introduction

T-cell acute lymphoblastic leukemia (T-ALL) is an aggressive neoplasm of T-cell progenitors that accounts for ∼15% and 25% of pediatric and adult ALL cases, respectively.^1^ Although in pediatric T-ALL the introduction of high-dose, multiagent chemotherapy regimens has resulted in long-term event-free survival (EFS) rates of up to 90%,^2^ this survival benefit has not carried over to the adult T-ALL population, which have 5-year EFS rates of 40% to 50%. Treatment approaches for adult patients with relapsed or primary refractory T-ALL may include nelarabine or cytarabine-based salvage therapy coupled with bone marrow (BM) transplantation,^3^^,^^4^ but outcomes remain dismal with a median survival of 5 months.^4^ The differences in treatment outcomes are likely related to underlying genetic differences between children and adults. The search for molecular drug targets and the design of tumor-specific therapies is necessary to improve outcomes and reduce toxicity for high-risk adult patients with T-ALL.

In T-ALL, the accumulation of genomic abnormalities leads to the aberrant expression of select transcription factors and activation of signaling pathways that result in increased proliferation, cell survival, and impaired differentiation of T-cell progenitors, which give rise to this disease.^5^^,^^6^ The NOTCH signaling pathway is essential for T-lymphopoiesis and T-lineage differentiation from thymic precursors.^7^^,^^8^ Moreover, *NOTCH1* gain-of-function mutations are the major genetic driver of T-ALL in adults (>60% of cases)^9^ and mouse models have demonstrated the potency for these mutations in T-ALL development.^10^^,^^11^ Recent genomic studies have also identified recurrent mutations in epigenetic regulators in patients with T-ALL,^12^^,^^13^ including the de novo DNA methyltransferase enzyme *DNMT3A*. Although *DNMT3A* mutations are rare in pediatric and adolescent T-ALL,^14^ they occur in 10% to 18% of adult cases and are associated with lower remission rates, higher incidence of relapse, and markedly poorer EFS and overall survival.^15^, ^16^, ^17^, ^18^ The spectrum of *DNMT3A* mutations in T-ALL is distinct from that seen in myeloid neoplasms (eg, less prevalence of *DNMT3A*^R882^ dominant negative mutations), implicating divergent mechanisms of leukemogenesis. Although *DNMT3A* mutations in acute myeloid leukemia (AML) and myelodysplastic syndromes are almost exclusively heterozygous,^19^^,^^20^ patients with T-ALL can harbor homozygous or compound heterozygous *DNMT3A* mutations.^15^^,^^17^^,^^21^

*DNMT3A* loss-of-function mutations frequently co-occur with *NOTCH1* gain-of-function mutations in patients with T-ALL.^17^ We previously used a conditional knockout mouse model to uncover a tumor suppressor function for Dnmt3a in T-cell transformation.^22^ Transplantation of control and *Dnmt3a*-deficient BM progenitor cells transduced with a retrovirus expressing the NOTCH1 intracellular domain (NICD)^10^ revealed a much shorter latency to T-ALL in a *Dnmt3a*-deficient background.^22^ Mice deficient for *Dnmt3a* in hematopoiesis also showed an accumulation of T-cell progenitors in the thymus as a possible mechanism of premalignant disposition.^22^ This developmental block of *Dnmt3a*-null T-cell progenitors was associated with reduced apoptosis.^22^ To translate those findings to malignant hematopoiesis, we now show here that mouse *Dnmt3a*-null T-ALL cells are also resistant to apoptosis. This phenomenon is conserved in humans because samples from patients with primary T-ALL with *DNMT3A* mutations are resistant to cell death induced by certain chemotherapeutics. Mechanistically, we show this prosurvival phenotype is driven by enhanced JAK/STAT and identify *BIRC5* as a specific genetic dependency and therapeutic vulnerability of *DNMT3A*-mutant T-ALL cells. These data provide a critical first step toward novel target precision medicine approaches for this patient group.

## Methods

### Mice and transplantation

The institutional animal care and use committee at Washington University approved all animal procedures. Transplant recipient mice (C57Bl/6 CD45.1, The Jackson Laboratory, no. 002014) received a split dose of irradiation (11 Gy) ∼4 hours apart. Patient-derived xenograft (PDX) models were generated by transplanting cells from patients with primary T-ALL into 6- to 8-week-old NOD-scid IL2Rgamma^null^ (The Jackson Laboratory, no. 005557) via tail vein injection in a volume of 200 μL with 27-gauge U-100 insulin syringes (EasyTouch, no. 08496-2755-01). Ruxolitinib (RUX) was administered in chow formulation (2 g RUX/1 kg chow; Incyte, no. INCB018424).

### Human T-ALL samples

Samples from patients with T-ALL were obtained with written consent in accordance with the Declaration of Helsinki protocol. Because all patient samples were deidentified and the study team had no access to individual patient health information, the Washington University institutional review board and human research protection office determined this to be a nonhuman study. Deidentified samples were cultured in vitro in StemSpan SFEM II Media (StemCell Technologies, no. 09655) supplemented with penicillin-streptomycin (50 U/mL), human stem cell factor (50 ng/mL), human interleukin-2 (IL-2; 50 U/mL), and human IL-7 (10 ng/mL).

### Drug treatments

Approximately 1 × 10^5^ cells were seeded in 48-well cell culture plates together with the different drugs. Cells were incubated for 48hours in a 5% CO_2_ incubator at 37⁰C, then cell viability was assessed using annexin-V flow cytometry (Invitrogen, no. 509279) with Sytox blue cell stain (ThermoFisher, no. S34857). For YM155 (Sepantronium Bromide) treatment, 2 × 10^6^ primary human T-ALL cells were seeded in 6-well tissue culture plate wells and incubated with YM155 (Selleckchem, no. S1130) at a concentration of 1 μM for 48 hours, then assayed for viability as described above.

### Statistics

Student *t* test and 1-way, and 2-way analysis of variance were used for statistical comparisons as appropriate. Survival curves were analyzed using a Mantel-Cox log-rank test. Significance is indicated using the following convention: ∗*P* < .05, ∗∗*P* < .01, ∗∗∗*P* < .001, and ∗∗∗∗*P* < .0001. All graphs represent mean ± standard error of the mean.

## Results

### DNMT3A mutations are founding events in T-ALL and alter DNA methylation

**Figure 1.**
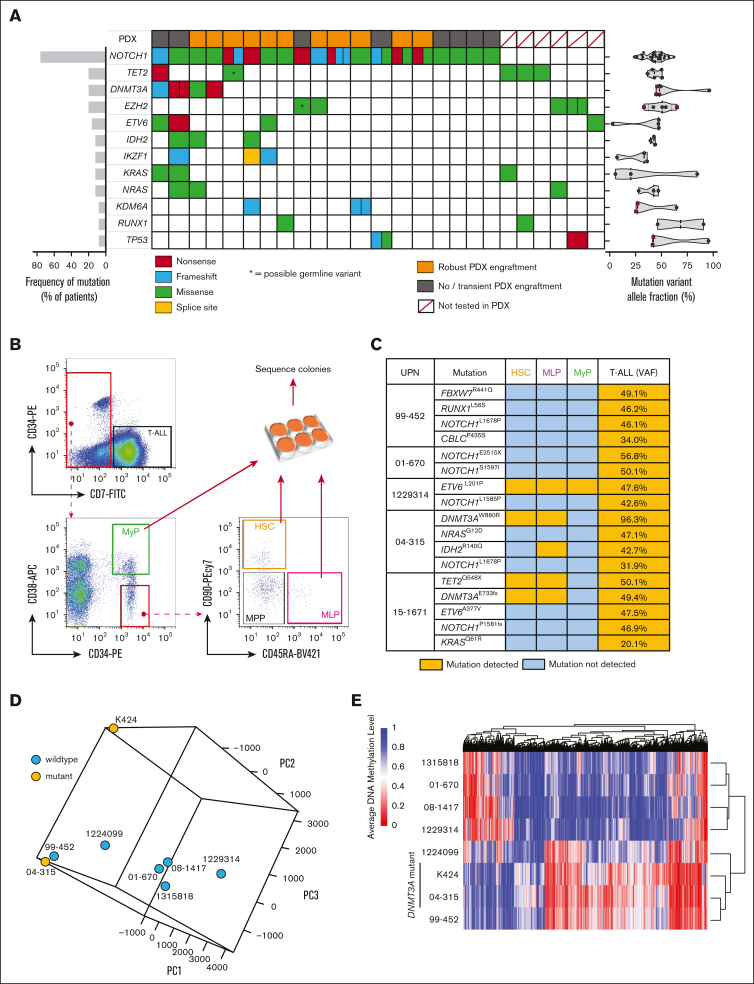
**DNMT3A mutations are founding events in T-ALL and alter DNA methylation.** (A) Genomic summary of the T-ALL cohort showing incidence of recurrently mutated genes, engraftment in PDXs, and VAFs. (B) Schematic for flow cytometric purification of HSPC populations from the BM of patients with T-ALL. (C) Summary of human HSPC-derived colony sequencing showing detection of different somatic variants in individual populations. (D) Principle component analysis of DNA methylation profiles of patients with T-ALL by whole-genome bisulfite sequencing. (E) Clustering of T-ALL samples based on differentially methylated regions.

We determined the mutational spectrum of a cohort of samples from 25 adult patients with T-ALL through targeted genomic sequencing (supplemental Table 1). As expected, *NOTCH1* variants were the predominant oncogenic lesion with 19 of 25 (76.0%) patients harboring mutations in *NOTCH1*, with 5 of 19 of these patients having multiple *NOTCH1* variants (Figure 1A). The next most recurrently mutated genes were the epigenetic regulators *TET2*, *DNMT3A*, and *EZH2* (Figure 1A). *DNMT3A* mutations co-occurred with *NOTCH1* mutations in this patient cohort as has been reported by other groups.^17^ The variant allele fraction (VAF) of *DNMT3A* mutations in patients with T-ALL was consistently ∼50% (Figure 1A), suggestive of heterozygous mutations found in all the tumor cells, presenting *DNMT3A* mutation as a founding event in T-ALL as in AML^23^ and myelodysplastic syndromes.^19^ As has previously been reported for T-ALL, 1 patient had compound *DNMT3A* mutations (*DNMT3A*^Q606X^ VAF = 45.9%; *DNMT3A*^W305X^ VAF = 44.8%). We attempted to establish PDXs to produce banks of primary cells for mechanistic study (Figure 1A; supplemental Figure 1A).

The VAF of *NOTCH1* mutations was highly variable, suggesting *NOTCH1* may act as a founding mutation or cooperating mutation in T-ALL, as has been suggested by recent studies.^24^^,^^25^ To definitively establish the ontogeny of mutations in T-ALL, hematopoietic stem and progenitor cell (HSPC) subsets were isolated from patient BM specimens by flow cytometry. Colonies were generated from hematopoietic stem cells (HSCs; CD7^−^ CD38^−^ CD34^+^ CD45RA^−^ CD90^+^), mixed lineage progenitors (CD7^−^ CD38^−^ CD34^+^ CD45RA^+^ CD90^−^), and myeloid progenitors (CD7^−^ CD38^+^ CD34^+^) for mutation sequencing analysis (Figure 1B). The abundance of these HSPCs (including multipotent progenitors; CD7^−^ CD38^−^ CD34^+^ CD45RA^−^ CD90^−^) in samples from patients with T-ALL were highly variable (supplemental Figure 1B-C). Targeted sequencing was performed for the mutations identified in bulk T-ALL cells in each of the HSPC populations (supplemental Figure 1D). *NOTCH1* mutations were never observed in HSPCs (Figure 1C). Conversely, the *DNMT3A* mutations identified in the bulk T-ALL for each patient were detected in HSPCs, indicating that they are an early event in T-ALL oncogenesis. This suggests that in these cases, T-ALL likely evolves from underlying clonal hematopoiesis (CH) from primitive HSPCs that have acquired *DNMT3A* mutations. Mutations in other genes commonly associated with CH were also observed in HSPC populations such as *TET2*, *IDH2*, and *ETV6* (Figure 1C).

Because DNMT3A is a DNA methyltransferase enzyme, DNA methylation profiles were examined in PDX-derived hCD45^+^hCD7^+^ T-ALL cells by whole-genome bisulfite sequencing. Principle component analysis did not strictly group the *DNMT3A*-mutant samples based on global DNA methylation patterns (Figure 1D). However, analysis of the most variable CpGs did stratify the *DNMT3A*-mutant patients together (supplemental Figure 2A). When *DNMT3A*-mutant samples were grouped together, analysis of differentially methylated regions further segregated these patients (Figure 1E) and identified hypomethylation at important loci implicated in leukemogenesis such as *TERT* and the *HOX* locus (supplemental Figure 2B-C). As in AML,^26^ it appears that *DNMT3A* mutations do not lead to massive alteration of the DNA methylome in patients with T-ALL (supplemental Table 2).

### Order of mutational acquisition in T-ALL development

**Figure 2.**
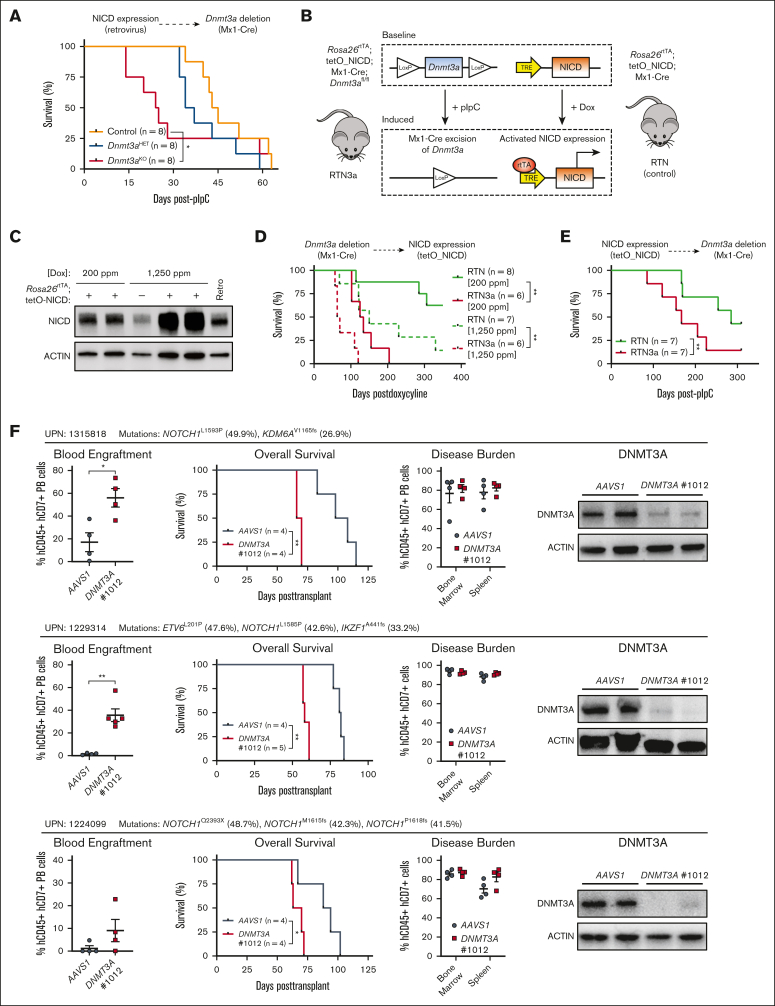
**Order of mutational acquisition in T-ALL development.** (A) Kaplan-Meier plot of mice that received transplantation with control, *Dnmt3a*^HET^-, and *Dnmt3a*^KO^-NICD–expressing cells and injected with pIpC 5 weeks after transplant. (B) Schematic for generation of 2-hit *Dnmt3a* loss-of-function *NICD* gain-of-function genetic mouse models. (C) Western blot showing expression levels of NICD from doxycycline-induced RTN mice with comparison with levels resulting from retroviral NICD expression. (D) Kaplan-Meier plot showing time to morbidity of RTN and RTN3a mice injected with pIpC and secondarily induced for NICD expression with doxycycline chow. (E) Kaplan-Meier plot showing time to morbidity of RTN and RTN3a mice induced for NICD expression with doxycycline chow then secondarily injected with pIpC. (F) Summary of in vivo *DNMT3A* CRISPR experiments with wild-type *DNMT3A* T-ALL specimens showing secondary transplant blood engraftment (6-8 weeks after transplant), time to morbidity, final disease burden at euthanasia, and protein levels of DNMT3A in T-ALL blasts at euthanasia.

Although *DNMT3A* mutations were found in HSPCs from patients with T-ALL, in some cases the VAF of *NOTCH1* mutations was higher than *DNMT3A* mutations in individual patients (supplemental Table 1). This brings into question the relevance of order of mutational acquisition in the pathological development of T-ALL. We previously used conditional knockout mice to elucidate a tumor suppressor function for Dnmt3a in T-cell transformation. Mx1-Cre:*Dnmt3a*^fl/+^ (*Dnmt3a*^HET^) and Mx1-Cre:*Dnmt3a*^fl/fl^ (*Dnmt3a*^KO^) along with Mx1-Cre:*Dnmt3a*^+/+^ (control) mice were treated with polyinosinic:polycytidylic acid (pIpC) to induce deletion of *Dnmt3a* in hematopoietic cells. BM progenitors were then transduced with a retrovirus expressing NICD, an oncogenic model that recapitulates many features of human T-ALL,^10^ and transplanted into recipient mice to reveal a much shorter latency to T-ALL development in a *Dnmt3a*-deficient background.^22^ We leveraged this model to test whether the order of mutational acquisition influences T-ALL development. BM progenitor cells from control, *Dnmt3a*^HET^, and *Dnmt3a*^KO^ mice were transduced with NICD retrovirus, and 500 transduced HSPCs (CD45.2^+^ green fluorescent protein [GFP]^+^ lineage^−^ Sca-1^+^ c-Kit^+^) were transplanted with a radioprotective dose of 3.0 × 10^5^ BM competitor cells. Once GFP^+^ engraftment reached an average of 10%, recipients were injected with pIpC to induce deletion of *Dnmt3a* after expression of NICD. Results showed that, as in our previous study, genetic deletion of *Dnmt3a* in an existing *Notch1*-mutant background also accelerated T-ALL development (Figure 2A).

Varying *NOTCH1* mutations in T-ALL drive downstream oncogenic signaling to various strengths,^11^ which can have important consequences for disease outcomes.^27^ Retroviral NICD leads to supraphysiological expression levels. To establish a more physiological model, we generated “2-hit” mice that allow independent induction of mutations, with *Dnmt3a* inactivation controlled through Cre/lox recombination and expression of NICD regulated by doxycycline induction (Figure 2B). Mx1-Cre:*Dnmt3a*^fl/fl^ mice were crossed to mice with the reverse tetracycline transactivator (rtTA) protein knocked in to the *Rosa26* locus (*Rosa26*^rtTA^), whereas NICD (amino acids 1749-2293) is expressed downstream of a tetracycline responsive element,^28^ acting as a “TetON” system. Level of NICD expression can be titrated by different concentrations of doxycycline in rodent chow (Figure 2C). We chose 200 ppm as a low dose to mimic “weak” *NOTCH1* mutations, and 1250 ppm as a high dose to model “strong” *NOTCH1* mutations.^11^ *Rosa26*^rtTA^; tetO_NICD; Mx1-Cre; *Dnmt3a*^+/+^ (RTN control) and *Rosa26*^rtTA^; tetO_NICD; Mx1-Cre; *Dnmt3a*^fl/fl^ (RTN3a) mice were treated with pIpC to inactivate *Dnmt3a*, and then 6-weeks later 2.0 × 10^6^ BM cells were transplanted into irradiated recipient mice. Doxycycline was administered 6-weeks after transplant through chow to activate NICD expression. Latency to T-ALL was reduced in RTN3a mice compared with control RTN mice regardless of doxycycline dose (Figure 2D). To reverse the mutation order in this model, 2.0 × 10^6^ BM cells from RTN and RTN3a mice were transplanted into irradiated mice. Recipients were placed on “low-dose” doxycycline chow 6-week after transplant to induce NICD expression, and then were injected with pIpC 10 weeks later. Consistent with all prior models, latency to T-ALL was accelerated in the absence of *Dnmt3a* (Figure 2E). Cumulatively, these data indicate that the order of *NOTCH1* and *DNMT3A* mutation acquisition is not important for the development of T-ALL, just that the 2 mutations be present in the same initiating cell.

To validate *DNMT3A* as a T-ALL tumor suppressor in human cells, guide RNAs (gRNAs) were designed to inactivate *DNMT3A* by CRISPR/CRISPR–associated protein 9 (CRISPR/Cas9) genome targeting in cells from patients with primary T-ALL with wild-type *DNMT3A*. Two previously validated gRNAs that target human *DNMT3A*^29^ were used alongside a negative control gRNA that targets the inert *AAVS1* locus. gRNA/Cas9 ribonucleoprotein complexes were nucleofected into primary human T-ALL cells and 48 hours after nucleofection, 2.5 × 10^5^ targeted cells were transplanted into NOD-scid IL2Rgamma^null^ mice. Generally, no significant differences were observed between *AAVS1* and *DNMT3A* gRNA-targeted groups across different patient samples as assessed by leukemic burden in the blood, BM, and spleen of moribund mice or time to morbidity (supplemental Figure 3). Analysis of VAFs showed that the frequency of *DNMT3A*-edited cells tended to increase over the course of the transplant (supplemental Figure 3), suggesting a potential competitive advantage. However, DNMT3A protein could still be detected in the leukemic blasts of moribund mice (supplemental Figure 3), suggesting that the timeframe of these experiments may not have been sufficient to observe dramatic differences in survival. Secondary transplantation was performed and, in this setting, *DNMT3A*-targeted cells showed much more rapid engraftment in the peripheral blood, significantly shorter time to morbidity, and almost complete elimination of DNMT3A protein (Figure 2F).

### JAK/STAT signaling is activated in DNMT3A-mutant T-ALL

**Figure 3.**
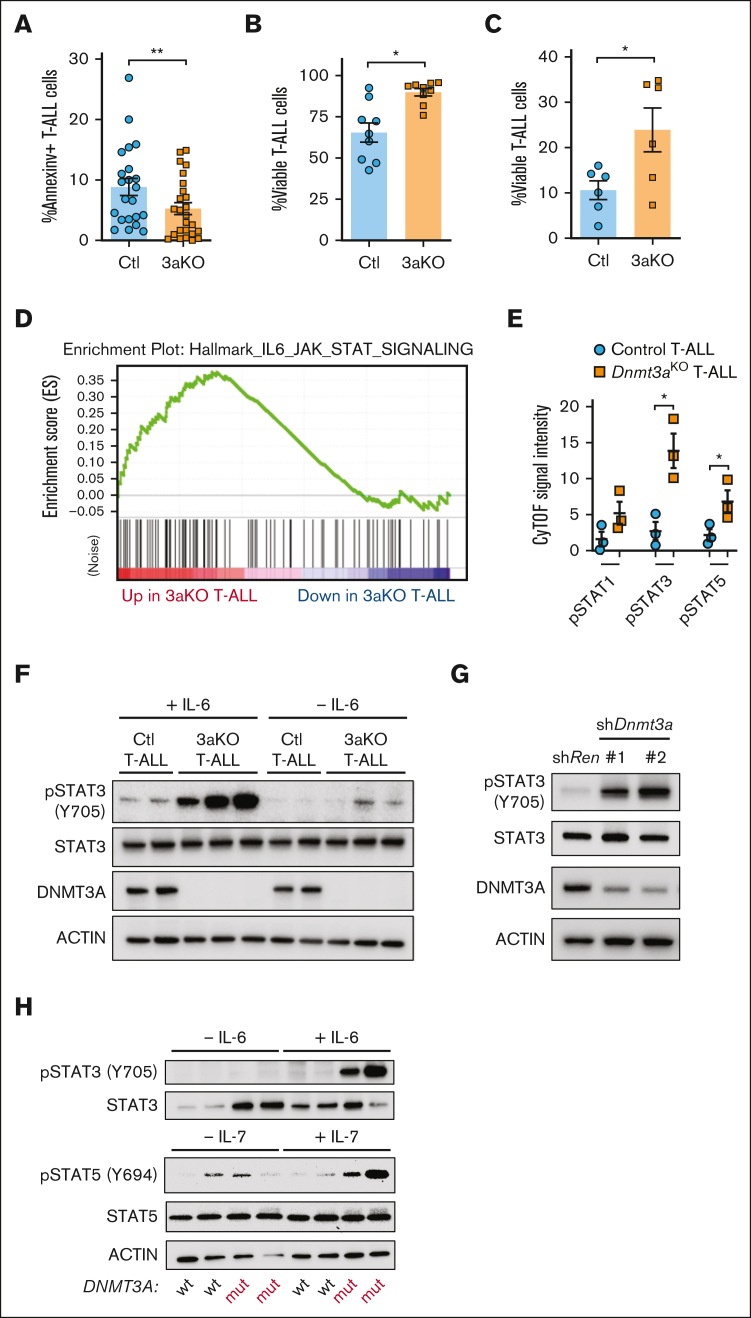
**JAK/STAT signaling in development of Dnmt3a-mutant T-ALL.** (A) Percentage of annexin-V^+^ (apoptotic) NICD-expressing control (Ctl) or *Dnmt3a*^KO^ (3aKO) T-ALL blasts freshly isolated from moribund recipient mice. (B) Proportion of viable T-ALL cells of indicated genotypes after 6-day in vitro coculture on OP9-DL1 cells. (C) Proportion of viable T-ALL cells of indicated genotypes after 6-day in vitro culture without stromal support. (D) GSEA plot showing enrichment of JAK/STAT gene expression in mouse *Dnmt3a*^KO^ T-ALL cells. (E) Cytometry by time of flight (CyTOF) analysis showing levels of phosphorylated Stat proteins in mouse T-ALL cells of indicated genotypes. (F) Western blot of control (Ctl) and *Dnmt3a*^KO^ (3aKO) T-ALL cells after in vitro stimulation with IL-6. (G) Western blot of MOHITO cells transduced with control (*Renilla*) or *Dnmt3a* short hairpin RNAs. (H) Western blot of human T-ALL cells after in vitro stimulation with IL-6 or IL-7.

We previously identified that loss of *Dnmt3a* in mouse T-cell progenitors lead to developmental arrest and reduced apoptosis.^22^ Annexin-V staining showed apoptosis was also lower in *Dnmt3a*^KO^ T-ALL cells (Figure 3A). With additional stress of a 6-day in vitro culture, *Dnmt3a*^KO^ T-ALL cells also showed a survival advantage with OP9-DL1 coculture (Figure 3B) or without stromal cell support (Figure 3C).

To identify transcriptional signatures associated with this prosurvival phenotype, we examined gene expression (RNA-sequencing) data comparing *Dnmt3a*^KO^ and control T-ALL blasts.^22^ Gene set enrichment analysis (GSEA; supplemental Figure 4) revealed 1 of the pathways most significantly different between the genotypes was JAK/STAT signaling, which was elevated in the *Dnmt3a*^KO^ background (Figure 3D). Cytometry by time of flight (CyTOF) mass cytometry identified higher levels of phosphorylated STAT proteins in *Dnmt3a*^KO^ T-ALLs (Figure 3E). Increased pSTAT3 was confirmed in freshly isolated leukemic *Dnmt3a*^KO^ T-ALL blasts, and *Dnmt3a*^KO^ T-ALL blasts were hypersensitive to IL-6 stimulation (Figure 3F). Introduction of short hairpin RNAs targeting *Dnmt3a* in Mouse Hematopoietic Interleukin-dependent cell line of T-cell Origin (MOHITO) cells (mouse IL-7–dependent CD4^+^CD8^+^ T-cell line) showed increased pSTAT3 (Figure 3G). This isogenic system suggests that increased JAK/STAT signaling is a direct effect of *Dnmt3a* loss-of-function in premalignant T-cells. Human T-ALL specimens with *DNMT3A* mutations also showed hypersensitive JAK/STAT signaling in response to cytokine stimulation (Figure 3H).

### JAK/STAT signaling in development of Dnmt3a-mutant T-ALL

**Figure 4.**
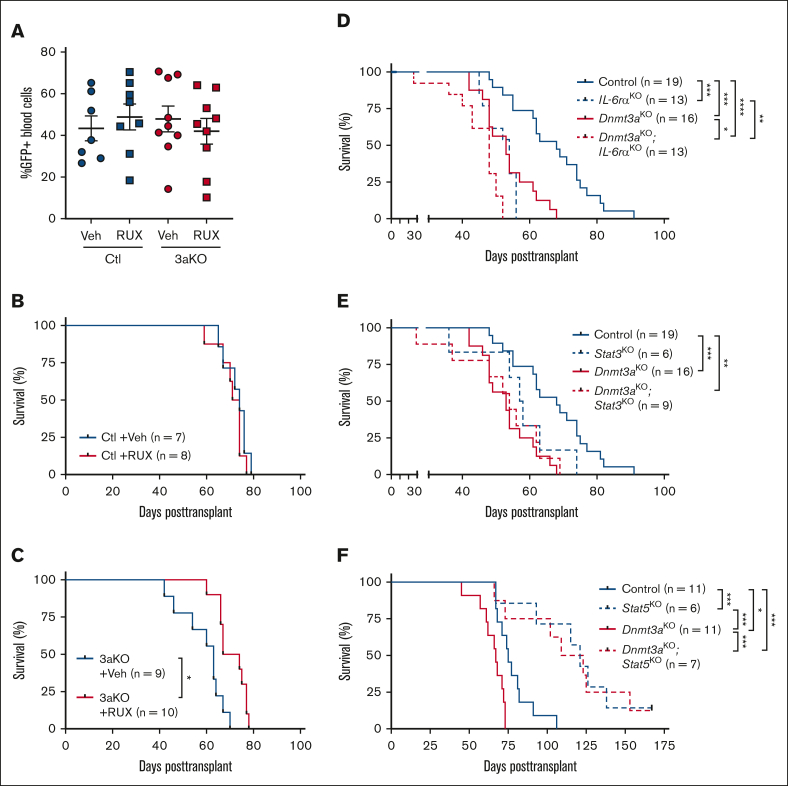
**JAK/STAT signaling in development of Dnmt3a-mutant T-ALL.** (A) Engraftment levels of NICD-expressing Control (Ctl) or *Dnmt3a*^KO^ (3aKO) T-ALL cells in recipient mice before treatment with RUX or vehicle (veh) chow. (B) Kaplan-Meier plot of mice transplanted with control NICD-expressing cells treated with RUX or vehicle (veh) chow. (C) Kaplan-Meier plot of mice transplanted with *Dnmt3a*^KO^ NICD-expressing cells treated with RUX or vehicle (veh) chow. (D) Kaplan-Meier plot of mice transplanted with NICD-expressing cells of indicated genotypes with and without *IL-6rα*. (E) Kaplan-Meier plot of mice transplanted with NICD-expressing cells of indicated genotypes with and without *Stat3*. (F) Kaplan-Meier plot of mice transplanted with NICD-expressing cells of indicated genotypes with and without *Stat5*.

Given the importance of JAK/STAT signaling in T-ALL^30^^,^^31^ and therapy resistance,^32^ we hypothesized that enhanced activity of this pathway might underlie the increased pathogenesis of *Dnmt3a*-mutant T-ALL. To determine if mitigation of this signaling pathway influenced *Dnmt3a*-mutant T-ALL, control and *Dnmt3a*^KO^ BM progenitor cells from pIpC-induced mice were transduced with NICD retrovirus and transplanted. Mice were randomized based on 5-week peripheral blood T-ALL burden (Figure 4A) to receive the JAK/STAT inhibitor RUX or vehicle control chow. Although RUX had no effect on development of control T-ALL (Figure 4B), mice engrafted with *Dnmt3a*^KO^ T-ALL showed a significant survival benefit with RUX treatment (Figure 4C).

To determine whether JAK/STAT signaling is also important for clonal expansion of nontransformed *Dnmt3a*-mutant HSCs, competitive HSC transplantation was performed. A total of 200 HSCs (CD45.2^+^ lineage^−^ Sca-1^+^ c-Kit^+^ CD48^−^ CD150^+^) from Vav-Cre:*Dnmt3a*^+/+^ (Control), Vav-Cre:*Dnmt3a*^fl/+^ (*Dnmt3a*^HET^), Vav-Cre:*Dnmt3a*^fl/fl^ (*Dnmt3a*^KO^), and Vav-Cre:*Dnmt3a*^R878H/+^ (*Dnmt3a*^R878^; mouse homolog of *DNMT3A*^R882H^) were transplanted into lethally irradiated mice in competition with 2.5 × 10^5^ congenic (CD45.1) BM cells. Recipient mice of each HSC genotype were randomized to receive either RUX or vehicle control chow between weeks 5 to 9 and 11 to 15 after transplant (with a 2-week drug “break” in the interval). RUX had no significant effect on donor-derived peripheral blood chimerism (supplemental Figure 5A), BM cellularity (supplemental Figure 5B), BM engraftment (supplemental Figure 5C), or self-renewal of donor-derived HSCs in the BM within each genotype (supplemental Figure 5D). To determine whether JAK/STAT inhibition had any long-term impact on HSC function, 200 donor-derived HSCs were purified from primary recipients and transferred into secondary hosts along with 2.5 × 10^5^ fresh competitor BM cells. In this setting, there were some genotype-specific differences. Prior exposure to RUX enhanced overall engraftment of *Dnmt3a*^HET^ cells in the peripheral blood and BM without affecting HSC self-renewal, whereas clonal expansion of *Dnmt3a*^KO^ HSCs was mitigated by prior JAK/STAT inhibition (supplemental Figure 5E-H). This suggests that JAK/STAT signaling may also regulate some of the phenotypes associated with *DNMT3A*-mutant CH.

Because *Dnmt3a*^KO^ T-ALL cells were hypersensitive to IL-6, and both pSTAT3 and pSTAT5 were elevated, we crossed mice with inactivating floxed alleles of *IL-6rα*,^33^ *Stat3*,^34^ and *Stat5*^35^ to Mx1-Cre:*Dnmt3a*^*fl/fl*^ mice. Resulting double-mutant strains were injected with pIpC to induce deletion of floxed alleles. After 6-weeks recovery, there were no significant differences in HSC numbers in the BM (supplemental Figure 6). NICD retroviral transduction and transplantation assays were performed as described above. Loss of *IL-6rα* and *Stat3* in a *Dnmt3a*^KO^ background did not rescue the latency to T-ALL (Figure 4D-E). In contrast, loss of *Stat5* significantly impeded development of T-ALL, however there was no genotype selectivity (Figure 4F). Thus, although JAK/STAT signaling inhibition can mitigate the survival advantage of *Dnmt3a*^KO^ T-ALL, it is likely other pathways are also involved.

### DNMT3A mutations in T-ALL convey a survival benefit and chemotherapy resistance

**Figure 5.**
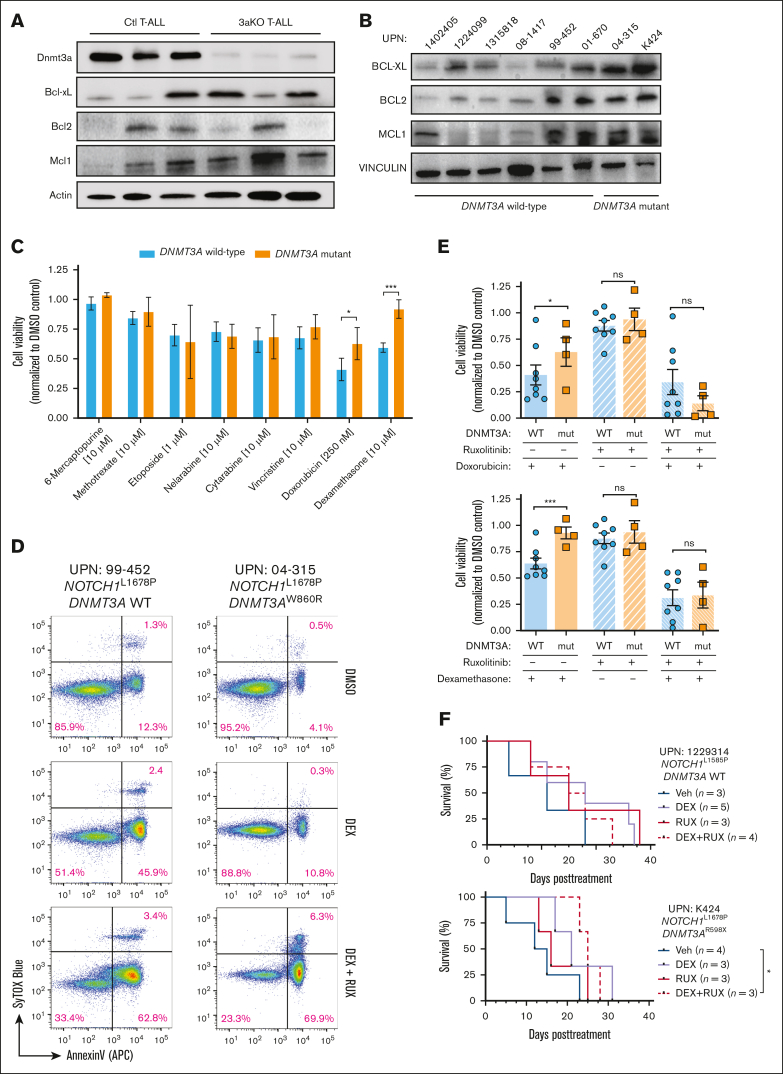
**DNMT3A mutations in T-ALL convey a survival benefit and chemotherapy resistance.** (A) Western blot of freshly isolated control (Ctl) and *Dnmt3a*^KO^ (3aKO) NICD-expressing T-ALL cells. (B) Western blot of primary human T-ALL cells showing expression of antiapoptotic proteins. (C) Cell viability of primary human T-ALL cells after 48 hours in vitro exposure to indicated chemotherapeutics normalized to DMSO control for each patient sample. (D) Representative flow cytometry plots showing annexin-V staining of T-ALL cells after exposure to DEX with/without RUX. (E) Cumulative cell viability of *DNMT3A* wild-type (WT) and mutant (mut) T-ALL cells after 48 hours in vitro exposure to indicated drug combinations normalized to DMSO control for each patient sample. (F) Kaplan-Meier plots of representative *DNMT3A* WT (UPN 1229314) and *DNMT3A* mutant (UPN K424) T-ALL specimens xenografted into NOD-scid IL2Rgamma^null^ (NSG) mice and then treated with indicated agents once T-ALL burden reached 10% of the peripheral blood.

JAK/STAT signaling can promote survival of T-ALL cells by upregulating antiapoptotic proteins. There was heterogeneous upregulation of BCL2 Apoptosis Regulator (BCL2), BCL2 Like 1 (BCL-xL), and MCL1 Apoptosis Regulator, BCL2 Family Member (MCL-1) in mouse (Figure 5A) and human (Figure 5B) T-ALL with *DNMT3A* mutations. We hypothesized apoptotic resistance may underlie the inferior response of patients with *DNMT3A*-mutant T-ALL to chemotherapy.^18^ Primary T-ALL specimens (Figure 1A) were subjected to a panel of agents that form the backbone of conventional T-ALL chemotherapy^36^; 48-hours later, cell viability was assessed. In general, *DNMT3A*-mutant T-ALL showed similar sensitivity to most chemotherapeutics compared with patient samples with wild-type *DNMT3A* but were resistant to apoptosis induced by doxorubicin and dexamethasone (DEX; Figure 5C). We repeated the drug treatment experiments in the presence of RUX, which resensitized cells from patients with *DNMT3A*-mutant T-ALL to apoptosis (Figure 5D), confirming JAK/STAT signaling potentiates the chemotherapy resistance phenotype of *DNMT3A*-mutant T-ALL (Figure 5E). To determine whether this may sensitize *DNMT3A*-mutant T-ALL cells in vivo, PDXs were established and treated with agents once human cell engraftment averaged 10%. Although treatment with DEX or RUX alone had no significant effect on *DNMT3A*-mutant T-ALL, combination therapy was able to significantly prolong survival of recipient mice (Figure 5F).

### BIRC5 supports the survival advantage of DNMT3A-mutant T-ALL cells

**Figure 6.**
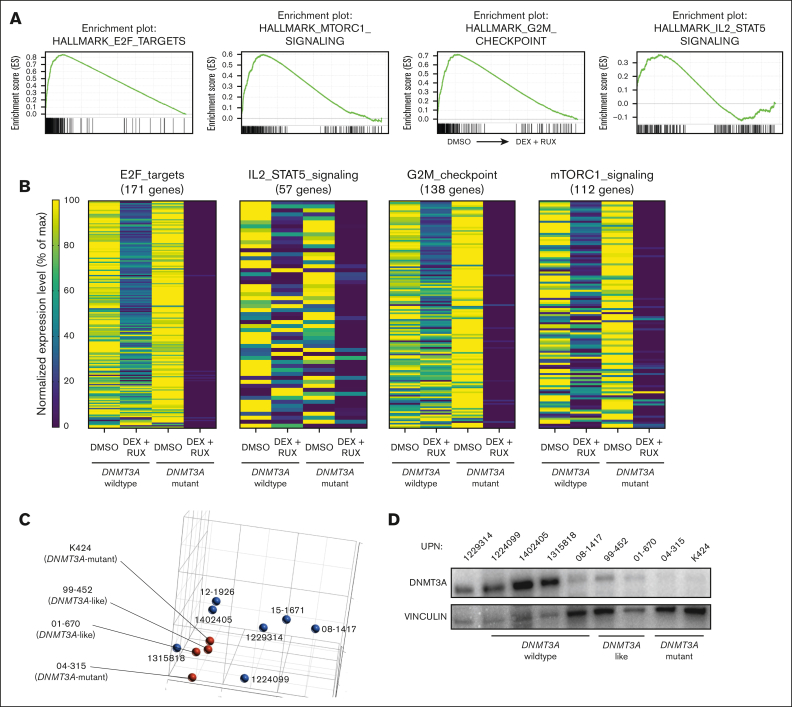

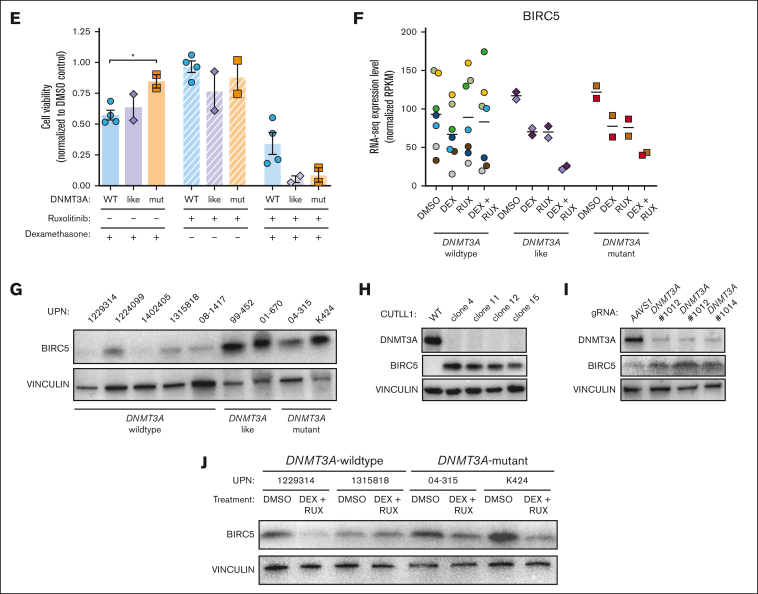
**BIRC5 supports the survival advantage of DNMT3A-mutant T-ALL cells.** (A) GSEA plots showing significantly different gene sets in *DNMT3A*-mutant T-ALL specimens after 24-hour treatment with DEX + RUX (all downregulated after treatment). (B) Expression levels of genes within significantly different gene sets comparing WT *DNMT3A* and *DNMT3A*-mutant samples after 24 hours exposure to indicated treatments. (C) Principle component analysis of DMSO-treated control T-ALL gene expression profiles from patients with T-ALL by RNA sequencing. (D) Western blot showing DNMT3A levels in indicated T-ALL specimens. (E) Cell viability of wild-type *DNMT3A* (WT), *DNMT3A*-mutant–like (like) and *DNMT3A*-mutant (mut) T-ALL cells after 48 hours in vitro exposure to indicated drug combinations normalized to DMSO control for each patient sample. (F) Normalized expression level of *BIRC5* in primary T-ALL cells after 24 hours exposure to indicated treatments. Individual patient specimens are denoted by the same color. (G) Western blot showing protein levels of BIRC5 in indicated samples from patients with T-ALL. (H) Western blot analysis of CUTTL1 cells after CRISPR/Cas9-mediated gene targeting of *DNMT3A*. (I) Western blot showing protein levels of BIRC5 in a representative WT *DNMT3A* T-ALL specimen after CRISPR/Cas9 targeting with indicated gRNAs. (J) Western blot for BIRC5 levels in specimens from patients with T-ALL after 48 hours treatment with indicated agents.

Glucocorticoids such as DEX play an essential role in the treatment of ALL and glucocorticoid resistance is an adverse prognostic factor.^37^ Activation of the JAK/STAT pathway is known to induce glucocorticoid resistance in patients with T-ALL.^30^^,^^32^ Our data suggest *DNMT3A* mutations may be a previously unrecognized mechanism that increases JAK/STAT signaling in T-ALL. To identify important genes and pathways that might support this phenotype, RNA-sequencing gene expression analysis was performed on T-ALL cells after 24 hours exposure to DEX (10 μM) in the presence or absence of the JAK/STAT inhibitor RUX (10 μM). GSEA of *DNMT3A*-mutant T-ALL samples comparing the dimethyl sulfoxide (DMSO) control with DEX + RUX treatment identified 4 differential gene sets: “*E2F targets I,*” “*MTORC1 signaling*,” “*G2M checkpoint*,” and “*IL2 / STAT5 signaling*”; all downregulated after DEX + RUX treatment (Figure 6A). Although some aspects of these gene sets were also downregulated in cells from patients with wild-type *DNMT3A* treated with these drugs, the magnitude was much greater in *DNMT3A*-mutant samples (Figure 6B). Principle component analysis of the control DMSO-treated cells to compare the global transcriptomes of the patient cells at baseline identified 2 *DNMT3A*-wild-type T-ALL samples that clustered with the *DNMT3A*-mutant samples (Figure 6C), which we designated “*DNMT3A*-mutant–like.” Western blot analysis showed patients with *DNMT3A*-mutant–like disease also had very low expression of DNMT3A, similar to patients with genetic mutations in *DNMT3A* (Figure 6D). DEX treatment of PDX-derived patient cells showed that *DNMT3A*-mutant–like samples were highly sensitive to the drug in the presence of RUX, comparable with cells from patients with *DNMT3A*-mutant T-ALL (Figure 6E). Thus, low DNMT3A expression in patients with T-ALL by heterogeneous mechanisms may confer drug resistance through JAK/STAT pathway activation.

One gene that was identified in multiple GSEA analyses was *BIRC5*. BIRC5 (also called Survivin) is a member of the inhibitor of apoptosis family of negative regulatory proteins that prevent apoptotic cell death.^38^ *BIRC5* was somewhat downregulated in patients with *DNMT3A*-mutant and *DNMT3A*-like T-ALL when treated with either DEX or RUX alone, but dramatically suppressed by the combination therapy, a trend not observed in patient samples without *DNMT3A* mutations (Figure 6F). Western blot confirmed the upregulation of BIRC5 in *DNMT3A*-mutant samples (Figure 6G). Because patients contain a heterogeneous mix of mutations, isogenic clones were created after CRISPR/Cas9 targeting of *DNMT3A* in the CUTTL1 T-ALL cell line. Western blot confirmed that elimination of *DNMT3A* coincided with increased BIRC5 (Figure 6H). Furthermore, BIRC5 was also heterogeneously increased in *DNMT3A*-wildtype T-ALL specimens after CRISPR/Cas9 targeting for *DNMT3A* (Figure 6I), and DEX + RUX treatment reduced BIRC5 protein levels (Figure 6J). Although the expression differences were not obviously linked to changes in DNA methylation (supplemental Figure 7), cumulatively these data show that *BIRC5* is increased in T-ALL cells as a direct consequence of *DNMT3A* loss of function.

### BIRC5 as a precision medicine target for DNMT3A-mutant T-ALL

**Figure 7.**
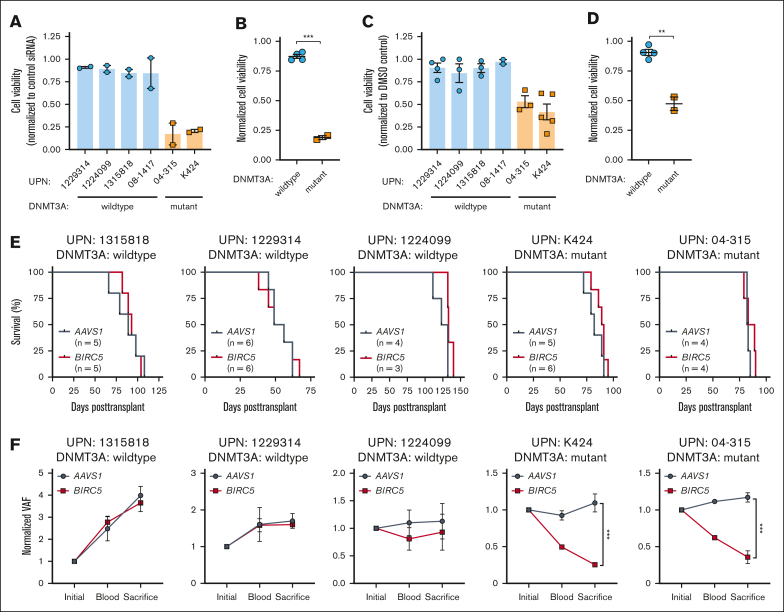
**BIRC5 as a precision medicine target for DNMT3A-mutant T-ALL.** (A) Cell viability of indicated T-ALL specimens (normalized to negative control small interfering [siRNA]) 48 hours after nucleofection with *BIRC5* siRNA. (B) Compiled normalized cell viability averaged for individual WT *DNMT3A* and *DNMT3A*-mutant T-ALL samples after nucleofection with *BIRC5* siRNA. (C) Cell viability of indicated T-ALL specimens (normalized to DMSO control) after 48 hours exposure to YM155. (D) Compiled normalized cell viability averaged for individual WT *DNMT3A* and *DNMT3A*-mutant T-ALL samples after 48 hours exposure to YM155. (E) Kaplan-Meier plots of NSG mice xenografted with indicated T-ALL specimens after CRISPR/Cas9 genome targeting with indicated gRNAs. (F) VAF of CRISPR edits from indicated gRNAs in T-ALL blasts from peripheral blood (6-8 weeks after transplant) and BM of moribund mice (euthanized) normalized to initial targeting efficiency 48 hours after nucleofection (initial) at time of transplant.

We hypothesized that BIRC5 might specifically support the prosurvival phenotype of *DNMT3A*-mutant T-ALL. Patient T-ALL cells were nucleofected with small interfering RNAs against human *BIRC5* (supplemental Figure 8A). Exposure of cells from patients with wild-type *DNMT3A* T-ALL to *BIRC5* small interfering RNA had minimal effect on cell viability (Figure 7A-B). In contrast, *BIRC5* inhibition induced significant apoptosis in *DNMT3A*-mutant T-ALL cells (Figure 7A-B). Sepantronium bromide (YM155) has been identified to inhibit BIRC5 expression at the messenger RNA and protein level.^39^ Treatment of JURKAT cells with YM155 inhibited BIRC5 expression (supplemental Figure 8B) and *DNMT3A*-knockout JURKAT cells showed increased sensitivity to YM155 (supplemental Figure 8C). A drug concentration gradient using primary T-ALL cells identified a dose that showed differential effects in *DNMT3A*-mutant cells (supplemental Figure 8D). Treatment of T-ALL cells with 1 μM YM155 for 48 hours revealed a dramatic decrease in viability of *DNMT3A*-mutant cells with minimal impact on wild-type *DNMT3A* cells (Figure 7C-D). To determine whether YM155 might synergize with JAK/STAT inhibition, primary cells were cultured for 48 hours with 10 μM DEX and 10 μM RUX with or without 1 μM YM155. There was no synergistic effect (supplemental Figure 8E), suggesting that BIRC5 is likely a direct target of DEX plus RUX inhibition in primary T-ALL cells.

To test the role of *BIRC5* in *DNMT3A*-mutant T-ALL in vivo, we targeted the locus for genetic inactivation using CRISPR/Cas9. Three gRNAs targeting human *BIRC5* were designed, and most efficacious gRNA used for in vivo studies (supplemental Figure 8F). Across samples from patients with T-ALL, either wild-type and mutant for *DNMT3A*, genetic inhibition of *BIRC5* did not alter overall time to morbidity (Figure 7E) or disease burden compared with *AAVS1*-targeted cells (supplemental Figure 8G). However, although samples from patients with wild-type *DNMT3A* were still able to expand in the absence of *BIRC5*, *BIRC5*-targeted *DNMT3A*-mutant cells were rapidly outcompeted in vivo (supplemental Figure 8H). This severe competitive disadvantage (Figure 7F) supports the hypothesis that *BIRC5* is a specific genetic dependency of *DNMT3A*-mutant T-ALL and targeting *BIRC5* in this subset of patients may be an effective precision medicine strategy.

## Discussion

In T-ALL, *DNMT3A* mutations frequently co-occur with *NOTCH1* mutations and predict poor clinical outcomes.^16^, ^17^, ^18^ Although this mutational profile represents a minor fraction of adult patients with T-ALL overall, it represents a major population of difficult-to-treat cases with poor outcomes. Our studies highlight new clinical approaches for these patients. Results here show that 1 of the major functional consequences of *DNMT3A* mutations in patients with T-ALL is to potentiate JAK/STAT signaling, leading to enhanced survival. Aberrant JAK/STAT signaling has previously been recognized as a potential pathogenic mechanism in T-ALL.^30^ The pathway is accentuated by activating genetic mutations in *IL7R*, *JAK1*, *JAK3*, and *STAT5* in 20% to 30% of T-ALL cases.^5^ Although clinical trials with RUX in relapsed/refractory acute leukemia have produced modest results, these trials did not consider genetics to select the most appropriate patient population that would benefit from such therapy. We propose that more appropriate selection of adult patients with T-ALL for JAK/STAT inhibition could produce significant clinical benefit. In addition to genetic mutations in *DNMT3A*, we show that DNMT3A suppression in T-ALL cells by alternative mechanisms (Figure 6D) also confers sensitivity to RUX (Figure 6E), potentially expanding the T-ALL population that may benefit from JAK/STAT inhibition. A complicating factor could arise from our observation that JAK/STAT inhibition may also have mutation-specific effects on underlying CH clones with *DNMT3A* mutations (supplemental Figure 5). However, genomic studies of patients with myelofibrosis treated long-term with RUX as standard of care has not identified clear selection biases of preleukemic clones with *DNMT3A* mutations.^40^, ^41^, ^42^

An unresolved question arising from these studies is how do *DNMT3A* mutations activate JAK/STAT signaling in T-ALL cells? We were unable to identify obvious DNA methylation alterations that could explain this (Figure 1E) and although expression of upstream regulators was increased in murine *Dnmt3a*^KO^ T-ALL cells (supplemental Figure 4B), levels of the core components was largely similar to control T-All cells, with the exception of *Ptpn6* (supplemental Figure 4C). *Ptpn6* encodes the tyrosine phosphatase SHP-1 that negatively regulates STAT signaling.^43^ It is possible that downregulation of *Ptpn6* leads to the inability to dephosphorylate activated STATs and potentiates the signaling in *Dnmt3a*^KO^ T-ALL cells. These mechanisms remain to be explored in future studies.

Mechanistically, our data show that *DNMT3A* loss of function upregulates *BIRC5* in T-ALL cells. BIRC5 inhibits cell death via both the extrinsic and intrinsic apoptotic pathways and suppresses caspase activity.^44^^,^^45^ It has been shown that inhibition of BIRC5 can induce tumor cell apoptosis and enhance sensitivity to chemotherapeutics or other apoptotic stimuli.^46^ Our data show that BIRC5 is upregulated in *DNMT3A*-mutant T-ALL specimens, and these patient cells are exquisitely sensitive to *BIRC5* inhibition. This positions *BIRC5* as a specific genetic dependency in *DNMT3A*-mutant T-ALL. Although there has been clinical interest in targeting this protein, there are currently no specific inhibitors available. Clinical efforts are ongoing to produce more specific BIRC5 inhibitors, and our results indicate that patients with *DNMT3A*-mutant T-ALL would be an ideal patient population to trial such agents.

Cumulatively, these studies reveal that *DNMT3A* mutations promote survival and chemotherapy resistance in cells from patients with T-ALL and identify *BIRC5* as a specific genetic dependency of *DNMT3A*-mutant T-ALL cells. These data provide a critical first step toward a novel target for precision medicine approaches for this patient group with poor outcomes.

Conflict-of-interest disclosure: A.L.Y. has performed consulting for BioGenerator; and is a cofounder, chief executive officer, and shareholder of Pairidex, Inc. T.M.P. has performed consulting for Pillar Patient Advocates, Silence Therapeutics, and the Myeloproliferative neoplasm research foundation (MPNRF). M.C.S. and H.C. are employees of Incyte Research Institute. G.A.C. has performed consulting and received research funding from Incyte, Ajax Therapeutics, and ReNAgade Therapeutics Management; and is a cofounder, member of the scientific advisory board, and shareholder of Pairidex, Inc. The remaining authors declare no competing financial interests.

The current affiliation for W.C.W. is DEM BioPharma, Cambridge, MA.

## Supplementary Material

Supplemental Table 3

Supplemental Figures, Legends for Tables, Methods, and References

## References

[1] Chiaretti S, Foa R (2009). T-cell acute lymphoblastic leukemia. Haematologica.

[2] Uckun FM, Gaynon PS, Sensel MG (1997). Clinical features and treatment outcome of childhood T-lineage acute lymphoblastic leukemia according to the apparent maturational stage of T-lineage leukemic blasts: a Children's Cancer Group study. J Clin Oncol.

[3] DeAngelo DJ, Yu D, Johnson JL (2007). Nelarabine induces complete remissions in adults with relapsed or refractory T-lineage acute lymphoblastic leukemia or lymphoblastic lymphoma: Cancer and Leukemia Group B study 19801. Blood.

[4] Thomas DA, Kantarjian H, Smith TL (1999). Primary refractory and relapsed adult acute lymphoblastic leukemia: characteristics, treatment results, and prognosis with salvage therapy. Cancer.

[5] Girardi T, Vicente C, Cools J, De Keersmaecker K (2017). The genetics and molecular biology of T-ALL. Blood.

[6] Bongiovanni D, Saccomani V, Piovan E (2017). Aberrant signaling pathways in T-cell acute lymphoblastic leukemia. Int J Mol Sci.

[7] Radtke F, Wilson A, Stark G (1999). Deficient T cell fate specification in mice with an induced inactivation of Notch1. Immunity.

[8] Pui JC, Allman D, Xu L (1999). Notch1 expression in early lymphopoiesis influences B versus T lineage determination. Immunity.

[9] Weng AP, Ferrando AA, Lee W (2004). Activating mutations of NOTCH1 in human T cell acute lymphoblastic leukemia. Science.

[10] Pear WS, Aster JC, Scott ML (1996). Exclusive development of T cell neoplasms in mice transplanted with bone marrow expressing activated Notch alleles. J Exp Med.

[11] Chiang MY, Xu L, Shestova O (2008). Leukemia-associated NOTCH1 alleles are weak tumor initiators but accelerate K-ras-initiated leukemia. J Clin Invest.

[12] Huether R, Dong L, Chen X (2014). The landscape of somatic mutations in epigenetic regulators across 1,000 paediatric cancer genomes. Nat Commun.

[13] Neumann M, Vosberg S, Schlee C (2015). Mutational spectrum of adult T-ALL. Oncotarget.

[14] Bond J, Touzart A, Lepretre S (2019). DNMT3A mutation is associated with increased age and adverse outcome in adult T-cell acute lymphoblastic leukemia. Haematologica.

[15] Simon C, Chagraoui J, Krosl J (2012). A key role for EZH2 and associated genes in mouse and human adult T-cell acute leukemia. Genes Dev.

[16] Aref S, El Menshawy N, El-Ghonemy MS, Zeid TA, El-Baiomy MA (2016). Clinicopathologic effect of DNMT3A mutation in adult T-cell acute lymphoblastic leukemia. Clin Lymphoma Myeloma Leuk.

[17] Grossmann V, Haferlach C, Weissmann S (2013). The molecular profile of adult T-cell acute lymphoblastic leukemia: mutations in RUNX1 and DNMT3A are associated with poor prognosis in T-ALL. Genes Chromosomes Cancer.

[18] Van Vlierberghe P, Ambesi-Impiombato A, De Keersmaecker K (2013). Prognostic relevance of integrated genetic profiling in adult T-cell acute lymphoblastic leukemia. Blood.

[19] Walter MJ, Ding L, Shen D (2011). Recurrent DNMT3A mutations in patients with myelodysplastic syndromes. Leukemia.

[20] Ley TJ, Ding L, Walter MJ (2010). DNMT3A mutations in acute myeloid leukemia. N Engl J Med.

[21] Van Vlierberghe P, Ambesi-Impiombato A, Perez-Garcia A (2011). ETV6 mutations in early immature human T cell leukemias. J Exp Med.

[22] Kramer AC, Kothari A, Wilson WC (2017). Dnmt3a regulates T-cell development and suppresses T-ALL transformation. Leukemia.

[23] Welch JS, Ley TJ, Link DC (2012). The origin and evolution of mutations in acute myeloid leukemia. Cell.

[24] Mansour MR, Duke V, Foroni L (2007). Notch-1 mutations are secondary events in some patients with T-cell acute lymphoblastic leukemia. Clin Cancer Res.

[25] Liu Y, Easton J, Shao Y (2017). The genomic landscape of pediatric and young adult T-lineage acute lymphoblastic leukemia. Nat Genet.

[26] Spencer DH, Russler-Germain DA, Ketkar S (2017). CpG Island hypermethylation mediated by DNMT3A is a consequence of AML progression. Cell.

[27] Issa N, Bjeije H, Wilson ER (2023). KDM6B protects T-ALL cells from NOTCH1-induced oncogenic stress. Leukemia.

[28] Stanger BZ, Datar R, Murtaugh LC, Melton DA (2005). Direct regulation of intestinal fate by Notch. Proc Natl Acad Sci U S A.

[29] Zhang CR, Ostrander EL, Kukhar O (2022). Txnip enhances fitness of Dnmt3a-mutant hematopoietic stem cells via p21. Blood Cancer Discov.

[30] Maude SL, Dolai S, Delgado-Martin C (2015). Efficacy of JAK/STAT pathway inhibition in murine xenograft models of early T-cell precursor (ETP) acute lymphoblastic leukemia. Blood.

[31] de Bock CE, Demeyer S, Degryse S (2018). HOXA9 cooperates with activated JAK/STAT signaling to drive leukemia development. Cancer Discov.

[32] Delgado-Martin C, Meyer LK, Huang BJ (2017). JAK/STAT pathway inhibition overcomes IL7-induced glucocorticoid resistance in a subset of human T-cell acute lymphoblastic leukemias. Leukemia.

[33] McFarland-Mancini MM, Funk HM, Paluch AM (2010). Differences in wound healing in mice with deficiency of IL-6 versus IL-6 receptor. J Immunol.

[34] Moh A, Iwamoto Y, Chai GX (2007). Role of STAT3 in liver regeneration: survival, DNA synthesis, inflammatory reaction and liver mass recovery. Lab Invest.

[35] Cui Y, Riedlinger G, Miyoshi K (2004). Inactivation of Stat5 in mouse mammary epithelium during pregnancy reveals distinct functions in cell proliferation, survival, and differentiation. Mol Cell Biol.

[36] Aries IM, Bodaar K, Karim SA (2018). PRC2 loss induces chemoresistance by repressing apoptosis in T cell acute lymphoblastic leukemia. J Exp Med.

[37] Pui CH, Evans WE (2006). Treatment of acute lymphoblastic leukemia. N Engl J Med.

[38] Deveraux QL, Reed JC (1999). IAP family proteins--suppressors of apoptosis. Genes Dev.

[39] Cheng Q, Ling X, Haller A (2012). Suppression of survivin promoter activity by YM155 involves disruption of Sp1-DNA interaction in the survivin core promoter. Int J Biochem Mol Biol.

[40] Patel KP, Newberry KJ, Luthra R (2015). Correlation of mutation profile and response in patients with myelofibrosis treated with ruxolitinib. Blood.

[41] Spiegel JY, McNamara C, Kennedy JA (2017). Impact of genomic alterations on outcomes in myelofibrosis patients undergoing JAK1/2 inhibitor therapy. Blood Adv.

[42] Tefferi A, Guglielmelli P, Lasho TL (2018). MIPSS70+ version 2.0: mutation and karyotype-enhanced International Prognostic Scoring System for primary myelofibrosis. J Clin Oncol.

[43] Demosthenous C, Han JJ, Hu G, Stenson M, Gupta M (2015). Loss of function mutations in PTPN6 promote STAT3 deregulation via JAK3 kinase in diffuse large B-cell lymphoma. Oncotarget.

[44] Small S, Keerthivasan G, Huang Z, Gurbuxani S, Crispino JD (2010). Overexpression of survivin initiates hematologic malignancies in vivo. Leukemia.

[45] Ambrosini G, Adida C, Altieri DC (1997). A novel anti-apoptosis gene, survivin, expressed in cancer and lymphoma. Nat Med.

[46] Altieri DC (2003). Validating survivin as a cancer therapeutic target. Nat Rev Cancer.

