# Supplementary material for: BIRC5 upregulation enhances DNMT3A-mutant T-ALL cell survival and pathogenesis
Source: Blood Neoplasia. 2024 Sep 5;1(4):100040. doi: 10.1016/j.bneo.2024.100040 (PMC12182843; doi:10.1016/j.bneo.2024.100040)
Supplement: Supplemental Figures, Legends for Tables, Methods, and References [file BNEO_NEO-2024-000314-mmc4.pdf]

BIRC5 Upregulation Enhances DNMT3A-Mutant T-ALL Cell Survival and Pathogenesis

Wangisa M.B. Dunuwille[1], W. Casey Wilson[1#], Hassan Bjeije[1], Nancy Issa[1], Wentao Han[1], Tyler M. Parsons[1], Andrew L. Young[2], Infencia Xavier Raj[1], Aishwarya Krishnan[1], Tarang Gaur[1], Eunice S. Wang[2], Andrew P. Weng[3], Matthew C. Stubbs[4], Hamza Celik[4], Amanda F. Cashen[1], John R. Edwards[5] and Grant A. Challen[1]*

[1] Division of Oncology, Department of Medicine, Washington University School of Medicine, St. Louis, MO, USA, 63110.

[2] Division of Hematology, Department of Medicine, Washington University School of Medicine, St. Louis, MO, USA, 63110.

[3] Roswell Park Comprehensive Cancer Center, Buffalo, NY 14263.

[4] Terry Fox Laboratory, BC Cancer Agency, Vancouver, BC, Canada.

[5] Incyte Research Institute, Wilmington, DE, USA, 19803.

[6] Center for Pharmacogenomics, Department of Medicine, Washington University School of Medicine, St. Louis, MO, USA, 63110.

[#] Current address: DEM BioPharma, Cambridge, MA 02139

[*]Corresponding author: Grant A. Challen, Ph.D.
Washington University School of Medicine
660 Euclid Avenue
St. Louis, MO, USA, 63110
Ph: +1 314-362-0987
Email: grantchallen@wustl.edu

LIST OF SUPPLEMENTARY MATERIALS

Fig. S1: ***T-ALL Patient Characterization***

Fig. S2: ***DNA Methylation Profiles of T-ALL Patients***

Fig. S3: ***DNMT3A CRISPR Targeting in DNMT3A wildtype T-ALL Patients Samples***

Fig. S4: ***JAK/STAT Pathway Activation in Dnmt3a-mutant Murine T-ALL***

Fig. S5: ***Effects of JAK/STAT Inhibition on Dnmt3a-Mutant HSCs***

Fig. S6: ***HSC Analysis of Donor Mice***

Fig. S7: ***DNA Methylation Profile of BIRC5 in T-ALL Patient Cells***

Fig. S8: ***BIRC5 as a Precision Medicine Target in DNMT3A-Mutant T-ALL Patients***

Table S1: ***Description of T-ALL Patients in This Study***

Summary of recurrently mutated genes identified in T-ALL patient specimens.

Table S2: ***DNA Methylation Profiles of T-ALL Patients***

Differentially methylated regions (DMRs) comparing *DNMT3A*-wildtype versus *DNMT3A*-mutant T-ALL patient samples.

Table S3: ***Gene Expression Profiles of T-ALL Patients***

Normalized gene expression values (RNA-seq RPKM) of T-ALL patient samples after 24-hour treatment with indicated agents. DEX = dexamethasone, RUX = ruxolitinib.

Supplemental Methods

Supplemental References

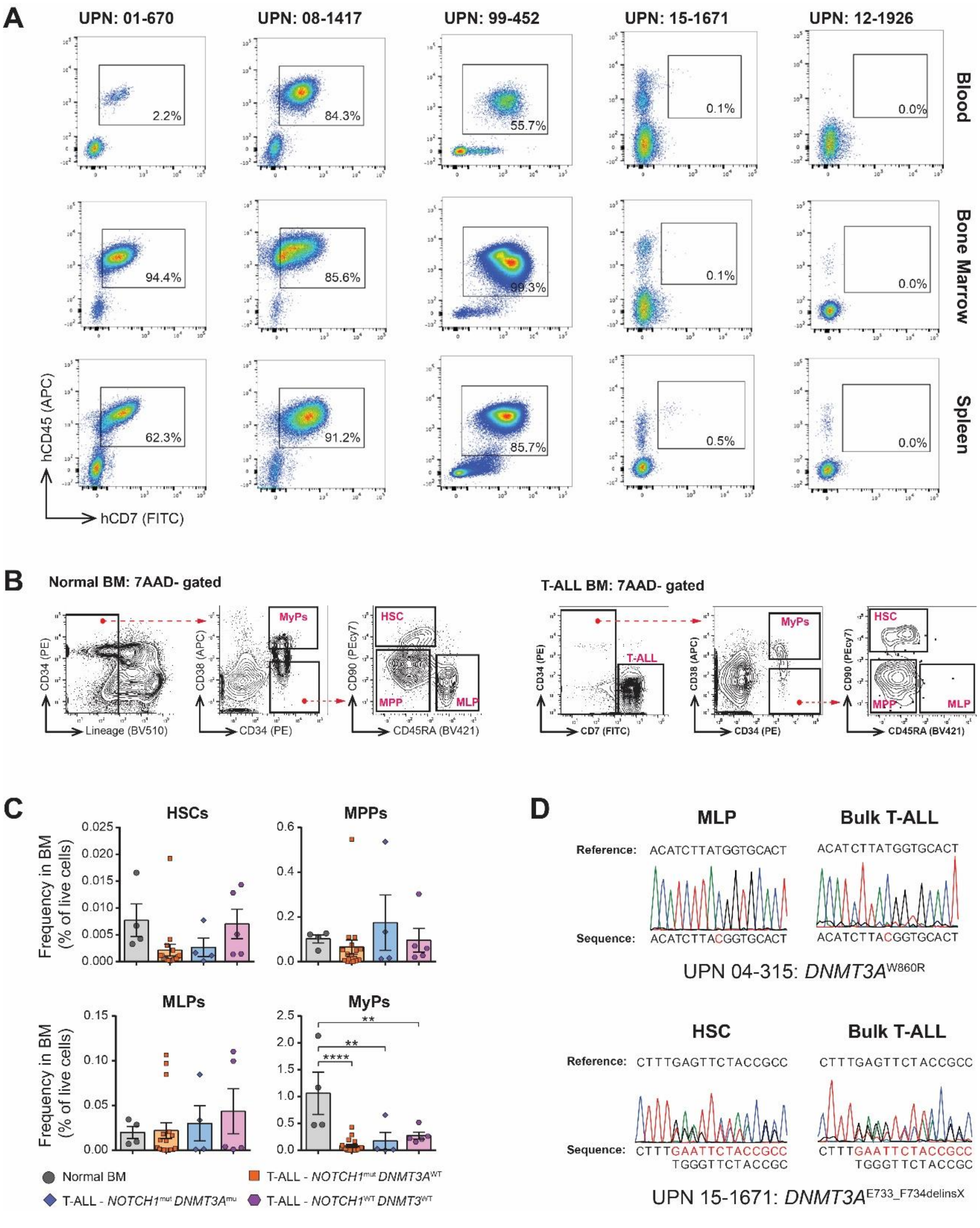

Fig. S1: *T-ALL Patient Characterization*

(**A**) Representative flow cytometry plots showing engraftment (or lack thereof) of T-ALL patient specimens in NSG mice. (**B**) Representative flow cytometry plots showing identification of HSPC populations in normal human bone marrow and T-ALL patient BM – MyPs = myeloid progenitors, HSC = hematopoietic stem cells, MPP = multipotent progenitor cells, MLP = mixed lineage progenitors. (**C**) Relative cell frequency of HSPC populations in normal human BM and T-ALL patient BM samples. (**D**) Sequencing traces showing identification of mutations found in bulk T-ALL samples in purified HSPC populations from T-ALL patient BM.

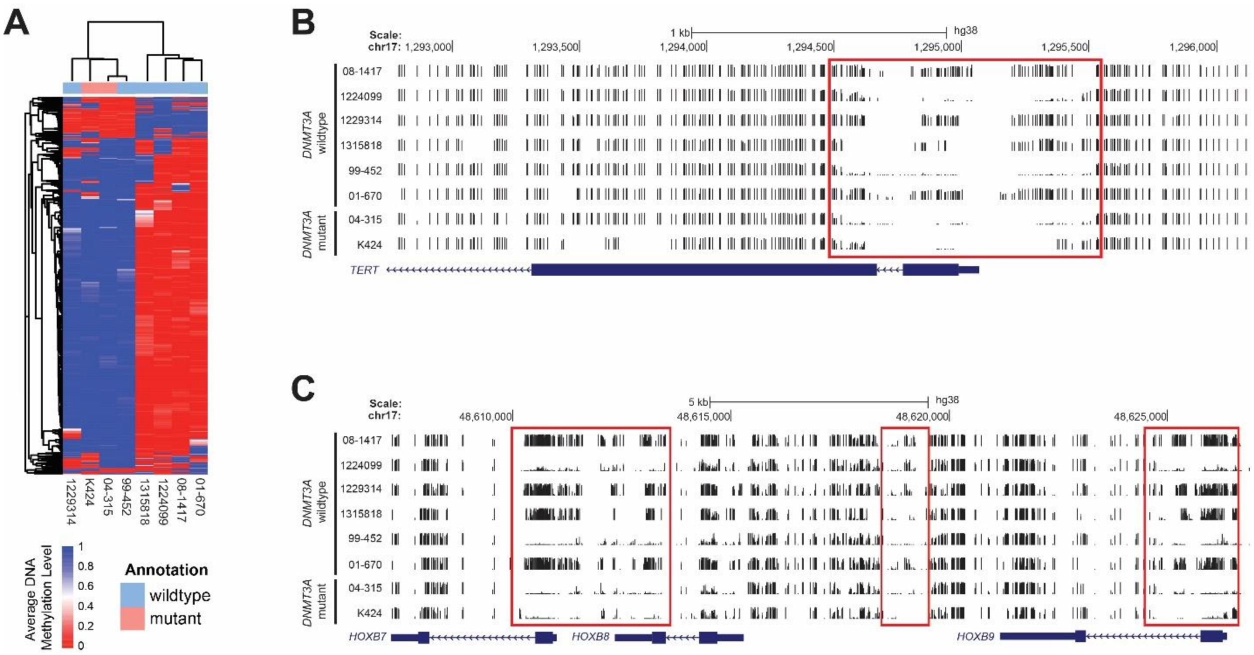

Fig. S2: ***DNA Methylation Profiles of T-ALL Patients***

(**A**) Clustering of T-ALL patients by DNA methylation profile based on the 10,000 most variable CpGs. (**B**) WGBS genome browser tracks showing DNA methylation levels of *TERT* promoter in T-ALL patient specimens. Height of each bar represents average DNA methylation level of individual CpGs. Red boxes denote hypomethylated DMRs in *DNMT3A* mutant patient samples. (**C**) WGBS genome browser tracks showing DNA methylation levels of *HOXB* locus in indicated T-ALL patient specimens. Height of each bar represents average DNA methylation level of individual CpGs. Red boxes denote hypomethylated DMRs in *DNMT3A* mutant patient samples.

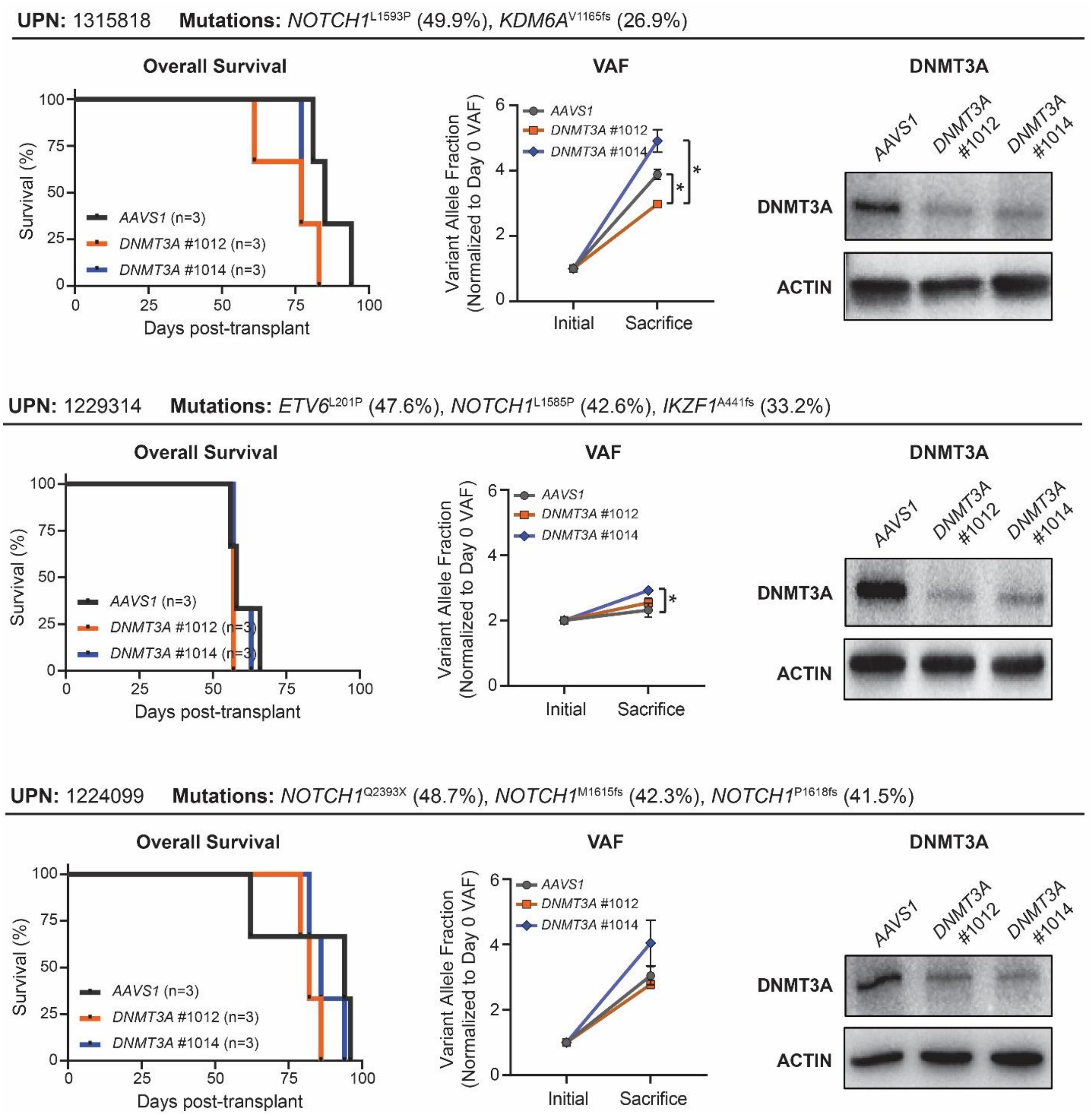

Fig. S3: ***DNMT3A CRISPR Targeting in DNMT3A wildtype T-ALL Patients Samples***

Summary of *in vivo DNMT3A* CRISPR experiments with *DNMT3A*-wildtype T-ALL patient specimens showing primary transplant time to morbidity, VAF of CRISPR edits from indicated gRNAs in T-ALL blasts from bone marrow of moribund mice (sacrifice) normalized to initial targeting efficiency 48-hours post-nucleofection (initial) at time of transplant, and protein levels of DNMT3A in T-ALL blasts at sacrifice.

A

Enriched in WT T-ALL

NAME	SIZE	NES	FDR q-val
HALLMARK_MYC_TARGETS_V1	186	2.79	0.0000
HALLMARK_OXIDATIVE_PHOSPHORYLATION	189	2.67	0.0000
HALLMARK_MYC_TARGETS_V2	56	2.28	0.0000
HALLMARK_DNA_REPAIR	132	1.82	0.0028
HALLMARK_E2F_TARGETS	191	1.70	0.0101
HALLMARK_FATTY_ACID_METABOLISM	122	1.61	0.0178
HALLMARK_MTORC1_SIGNALING	180	1.53	0.0269
HALLMARK_ADIPOGENESIS	165	1.47	0.0369
HALLMARK_UNFOLDED_PROTEIN_RESPONSE	96	1.42	0.0469

Enriched in KO T-ALL

NAME	SIZE	NES	FDR q-val
HALLMARK_HEME_METABOLISM	169	-1.809	0.0083
HALLMARK_COMPLEMENT	136	-1.789	0.0046
HALLMARK_COAGULATION	63	-1.765	0.0044
HALLMARK_MITOTIC_SPINDLE	177	-1.759	0.0035
HALLMARK_EPITHELIAL_MESENCHYMAL_TRANSITION	79	-1.758	0.0028
HALLMARK_INFLAMMATORY_RESPONSE	116	-1.380	0.0317
HALLMARK_IL6_JAK_STAT3_SIGNALING	62	-1.325	0.0340

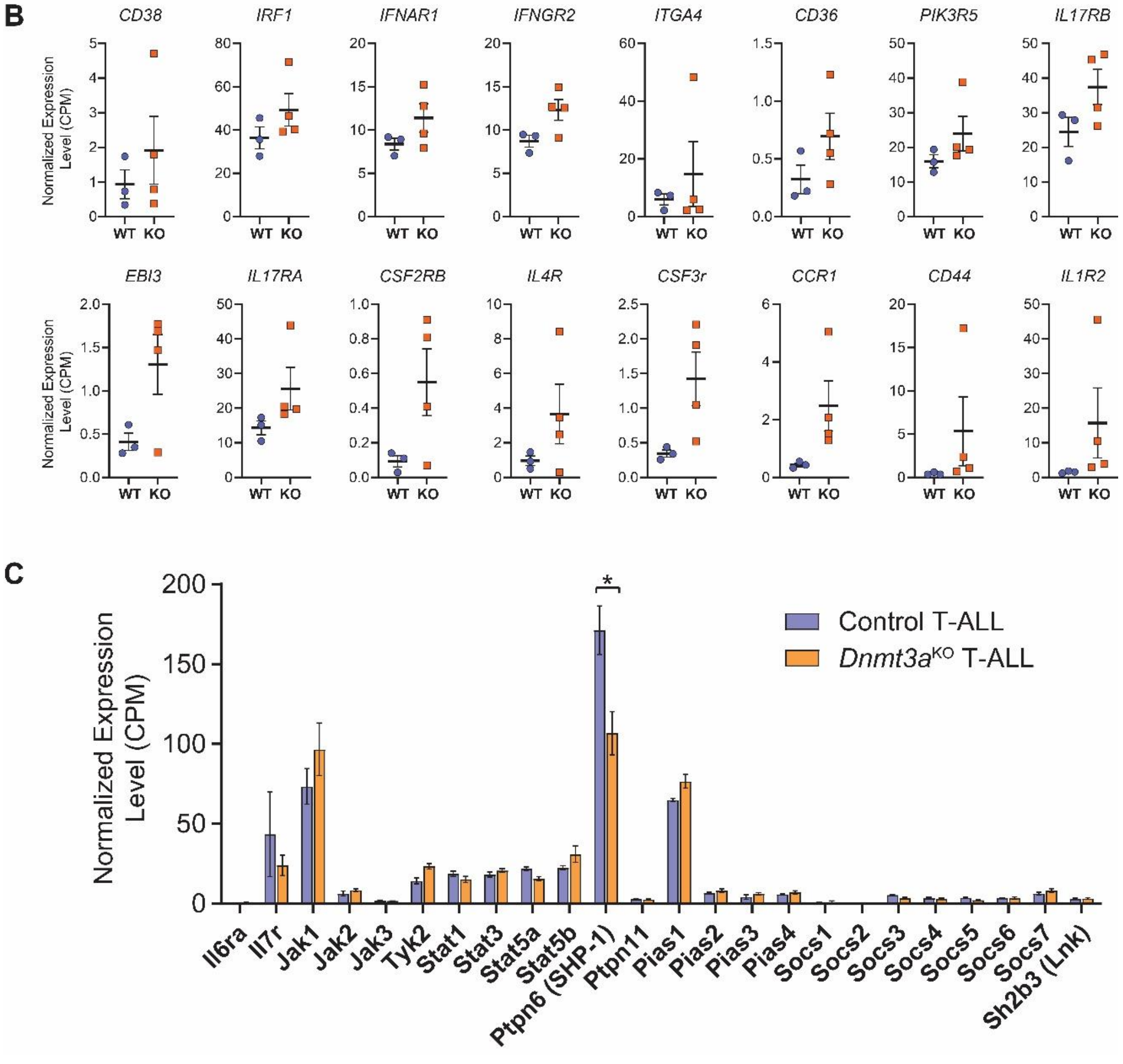

Fig. S4: *JAK/STAT Pathway Activation in Dnmt3a-mutant Murine T-ALL*

(**A**) Summary of genesets identified by GSEA as significantly different between control and *Dnmt3a*^{KO} murine T-ALL cells. (**B**) Expression levels of leading edge genes in "HALLMARK_IL6_STAT3_SIGNALING" geneset in control and *Dnmt3a*^{KO} murine T-ALL cells. (**C**) Expression levels of core JAK/STAT signaling genes in control and *Dnmt3a*^{KO} murine T-ALL cells. *Ptpn6* (SHP-1) is significantly downregulated in *Dnmt3a*^{KO} T-ALL cells

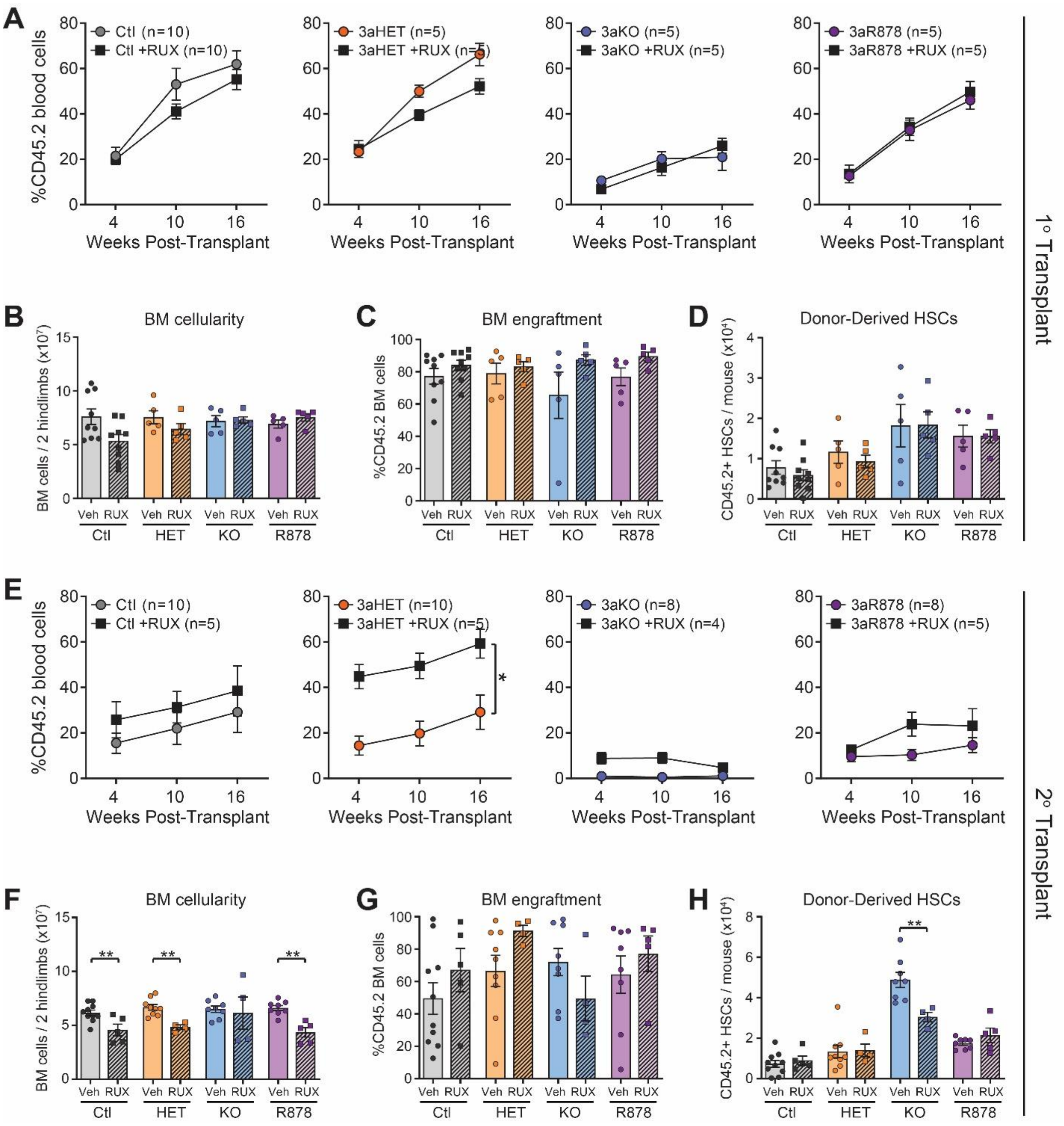

Fig. S5: ***Effects of JAK/STAT Inhibition on Dnmt3a-Mutant HSCs***

Primary transplant – (**A**) Peripheral blood engraftment of indicated HSC genotypes and treatments; (**B**) Total BM cellularity of recipient mice of indicated HSC genotypes and treatments 18-weeks post-transplant; (**C**) Bone marrow engraftment of indicated HSC genotypes and treatments 18-weeks post-transplant; (**D**) Absolute number of indicated HSC genotypes per recipient 18-weeks post-transplant. Secondary transplant - (**E**) Peripheral blood engraftment of indicated HSC genotypes and treatments; (**F**) Total BM cellularity of recipient mice of indicated HSC genotypes and treatments 18-weeks post-transplant; (**G**) Bone marrow engraftment of indicated HSC genotypes and treatments 18-weeks post-transplant; (**H**) Absolute number of indicated HSC genotypes per recipient 18-weeks post-transplant. Veh = vehicle, RUX = ruxolitinib, Ctl = control, 3aHET = *Dnmt3a*^{HET}, 3aKO = *Dnmt3a*^{KO}, 3aR878 = *Dnmt3a*$^{R878H.}$

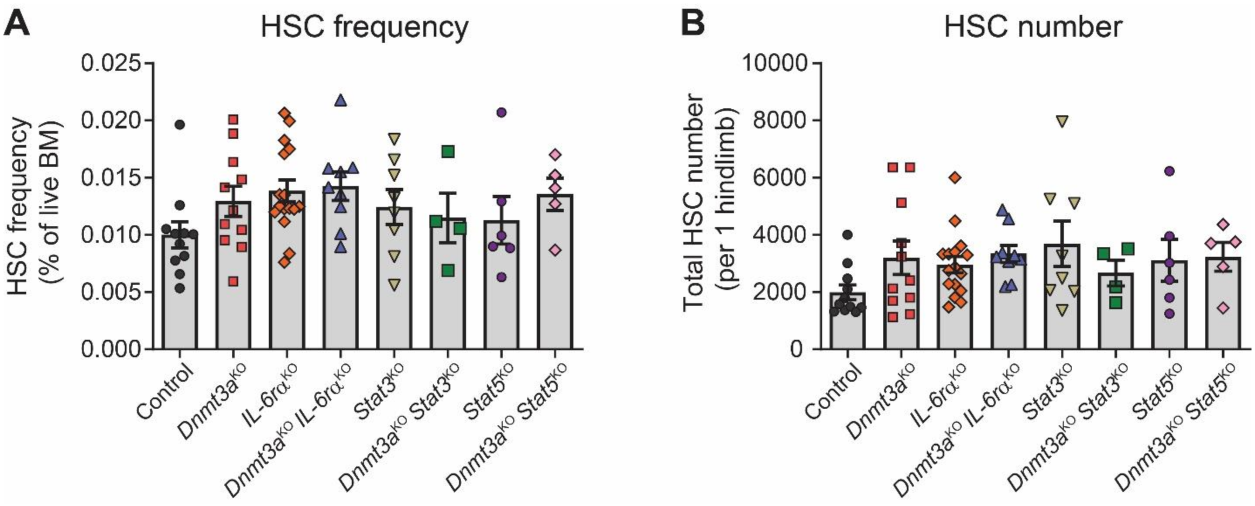

Fig. S6: ***HSC Analysis of Donor Mice***

(**A**) Frequency of HSCs (Lineage- Sca-1+ c-Kit+ CD48- CD150+) in BM of indicated mouse genotypes six-weeks post-induction with pIpC. (**B**) Absolute number of HSCs in BM of indicated mouse genotypes six-weeks post-induction with pIpC.

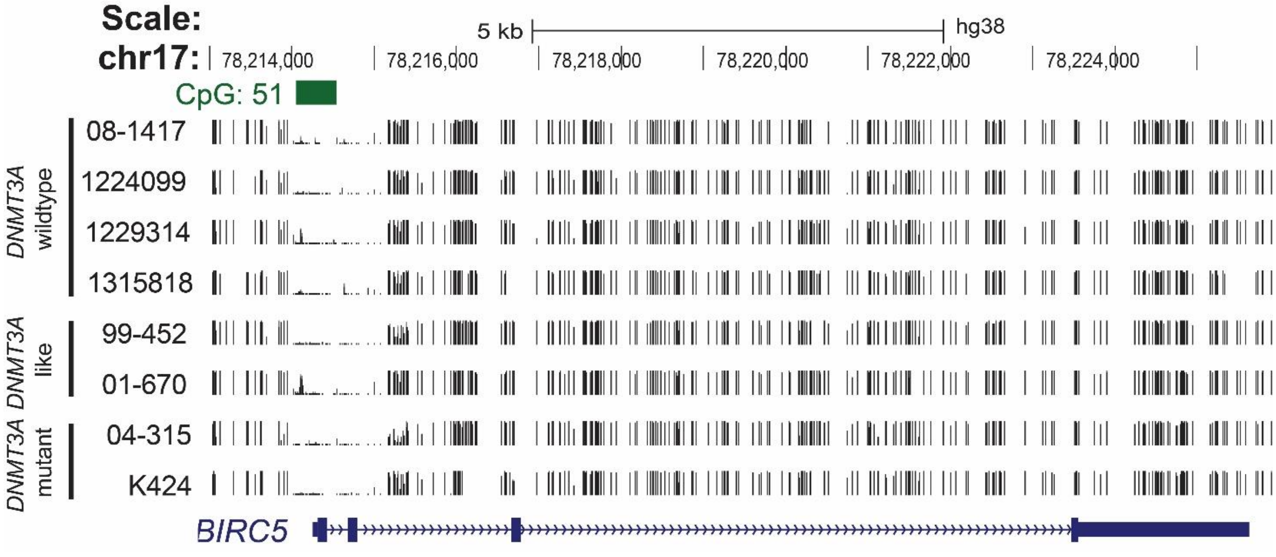

Fig. S7: ***BIRC5 as a Precision Medicine Target in DNMT3A-Mutant T-ALL Patients***

WGBS genome browser tracks showing DNA methylation levels of *BIRC5* locus in indicated T-ALL patient specimens. Height of each bar represents average DNA methylation level of individual CpGs.

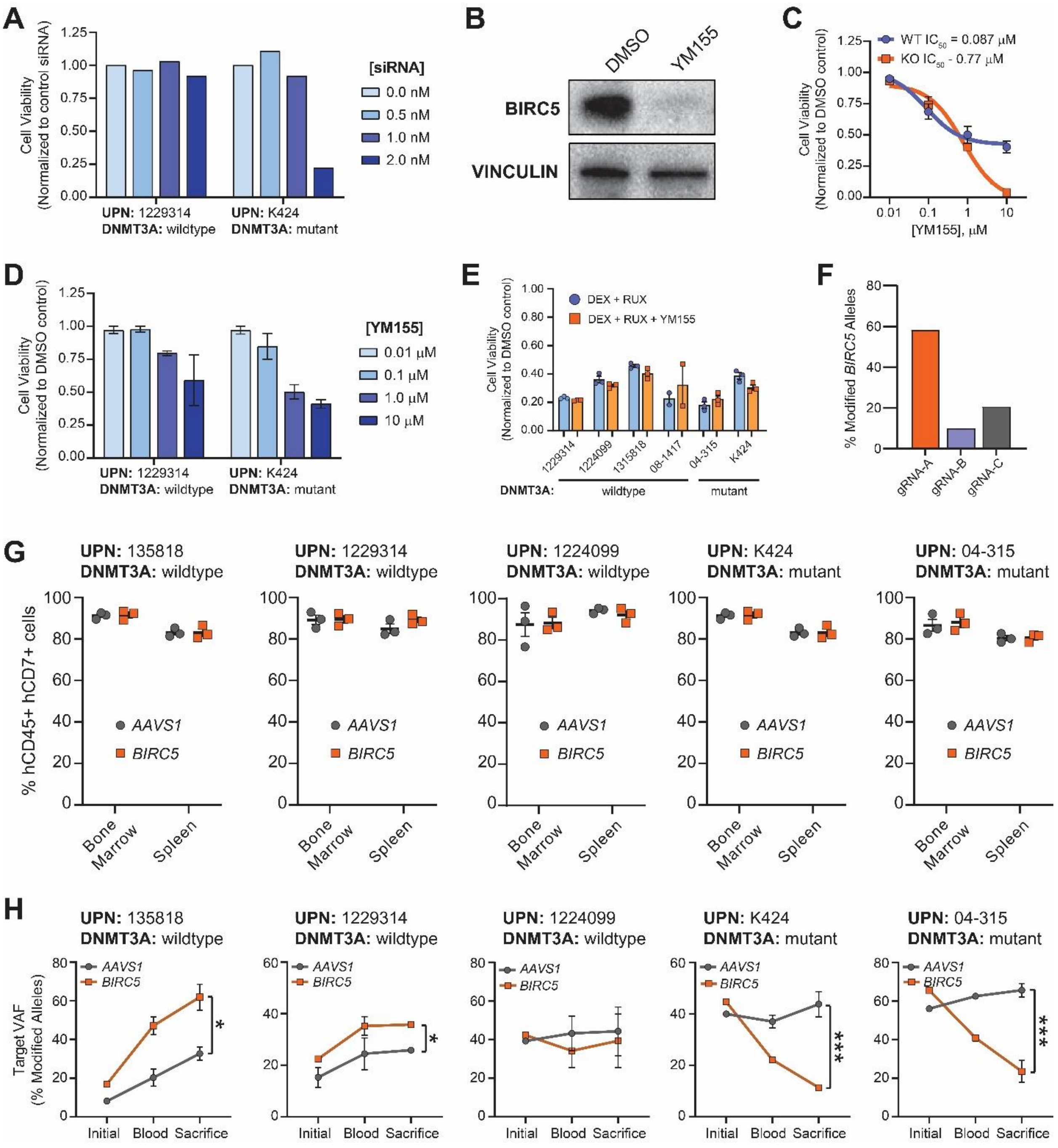

Fig. S8: *BIRC5 as a Precision Medicine Target in DNMT3A-Mutant T-ALL Patients*

(**A**) Cell viability of T-ALL patient specimens (normalized to negative control siRNA) 48-hours after nucleofection with increasing concentrations of *BIRC5* siRNA. (**B**) Western blot of JUIRKAT cells showing BIRC5 protein levels after 48-hour exposure to 10μM YM155. (**C**) Cell viability of wildtype (WT) and *DNMT3A*-knockout (KO) JURKAT cells with increasing concentrations of YM155. (**D**) Cell viability of T-ALL patient specimens (normalized to DMSO control) 48-hours after exposure to indicated concentrations of YM155. (**E**) Cell viability of indicated T-ALL patient specimens (normalized to DMSO control) after 48-hour exposure to DEX + RUX ± YM155. (**F**) CRISPR editing efficiency of gRNAs targeting *BIRC5* locus in JUIRKAT cells. gRNA-A was

chosen for functional studies in primary patient samples. (**G**) T-ALL disease burden at sacrifice in NSG mice receiving indicated T-ALL patient specimens targeted with indicated gRNAs. (**H**) Total VAF of CRISPR edits from indicated gRNAs in T-ALL blasts from peripheral blood (6-8 weeks post-transplant) and bone marrow of moribund mice (sacrifice) compared to initial targeting efficiency 48-hours post-nucleofection (initial) at time of transplant.

SUPPLEMENTAL METHODS

Mice and transplantation

The Institutional Animal Care and Use Committee at Washington University approved all animal procedures. For murine T-ALL experiments, all mice were C57Bl/6 background. *Dnmt3a*$^{fl/fl}$ [1], *Stat5*$^{fl/fl}$ (The Jackson Laboratory #002014), *Stat3*$^{fl/fl}$ (The Jackson Laboratory #002014) and *IL-6r*$\alpha^{fl/fl}$ (The Jackson Laboratory #012944) mice were crossed to Vav-Cre and Mx1-Cre strains. *Stat3*$^{fl/fl}$ mice were a kindly provided by Dr. Rajendra S. Apte (Washington University School of Medicine). To induce Mx1-Cre, six doses (300ug) of polyinosinic:polycytidylic acid (pIpC; Sigma #p1530) were administered every 48-hours via intraperitoneal injection to eight-week old mice. Mice were allowed to recover for six-weeks after the last pIpC injection prior to sacrifice for experimentation. Transplant recipients (C57Bl/6 CD45.1, The Jackson Laboratory #002014), received a split dose of irradiation (11 Gy) ~4 hours apart. Cells were transplanted via retro-orbital injection. "TetON" NICD mice were produced by crossing *Rosa26*^{rtTA} (The Jackson Laboratory #006965) mice with tetO_NICD mice [2], kindly supplied by Dr. Stacey Rentschler (Washington University School of Medicine).

Patient-derived xenograft (PDX) models were generated by transplanting primary T-ALL patient cells into 6–8 week-old NOD-scid IL2Rgamma^{null} (NSG; The Jackson Laboratory #005557) via tail vein injection in a volume of 200μL with 27-gauge U-100 insulin syringes (EasyTouch #08496-2755-01). Ruxolitinib was administered in chow formulation (2g ruxolitinib / 1kg chow; Incyte #INCB018424).

Plasmids and Viral Transduction

For production of retroviral particles, 293T cells (ATCC #CRL-3216) were co-transfected with packaging vector (pCL-Eco) and either empty vector control MSCV-IRES-GFP (MIG) or MIG-

NICD[3] using polyethylenimine (PEI; ThermoFisher Scientific #NC1014320). Viral supernatant was collected 48-hours post-transfection, centrifuged to remove cell debris, and stored at -80°C. For retroviral transduction, mice were injected with 5-flurouracil (150 mg/kg) six-days prior to experimentation. Sca-1+ bone marrow cells were purified using magnetic selection (Miltenyi Biotec) and plated in Stempro-34 medium (Gibco #10639011) supplemented with Pen/Strep (100 units/mL), L-glutamine (2 mM), mSCF (100 ng/mL), mTPO (100 ng/mL), mFlt3L (50 ng/ mL), mIL-3 (5 ng/mL), and polybrene (4 µg/mL; Sigma), and spinfected with retroviral supernatant at 250g for two-hours. Media was refreshed six-hours post-transduction, and cells were collected ~30-hours post-transduction for cell sorting and transplantation.

Human T-ALL Samples

Human T-ALL patient samples were obtained with written consent in accordance with the Declaration of Helsinki protocol. Because all patient samples were de-identified and the study team had no access to individual patient health information (PHI), the Washington University Institutional Review Board (IRB) and Human Research Protection Office (HRPO) determined this to be a non-human study. De-identified samples were cultured *in vitro* in StemSpan SFEM II Media (StemCell Technologies #09655) supplemented with Pen-Strep (50 U/mL), human stem cell factor (SCF; 50 ng/mL), human IL-2 (50U/mL), and human IL-7 (10 ng/mL).

Genome Sequencing

Genomic DNA was submitted for exome and/or targeted sequencing (Illumina TruSight Myeloid Sequencing Panel) at the McDonnell Genome Institute at Washington University. Sequence data was aligned to reference sequence build GRCh38 using BWA-mem (1) version 0.7.10 (params: -t 8), then merged and deduplicated using picard version 1.113. SNVs and indels, were detected using VarScan(2) version 2.4.2 (params: --min-coverage 8 --min-var-freq 0.1 –min-reads 2). Combined SNVs and indels were annotated by DoCM (Database of Curated Mutations,

params: --filter-docm-variants true), and further by Ensembl Variant Effect Predictor (VEP) of GRCh38 v95 (params: --coding-only false --everything --plugs [Downstream, Wildtype]) by providing gnomAD (The Genome Aggregation Database) and ClinVar VCF files. Variants were filtered by removing low quality variants (params: --min-base-quality 15, --min-mapping-quality 20), and removing sites that exceeded 0.1% population allele frequency in gnomAD projects.

Drug Treatments

A panel of chemotherapeutics that form the backbone of T-ALL therapy were used at previously published concentrations[4] to treat primary human cells *in vitro*. Approximately $1x10^5$ cells were seeded in 48-well cell culture plates together with the different drugs. Cells were incubated for 48-hours in a 5% CO_2 incubator at 37ºC, then cell viability was assessed using AnnexinV flow cytometry (Invitrogen #509279) with Sytox blue cell stain (ThermoFisher #S34857). For YM155 (Sepantronium Bromide) treatment, $2x10^6$ primary human T-ALL cells were seeded in six-well tissue culture plate wells and incubated with YM155 (Selleckchem #S1130) at a concentration of 1μM for 48-hours, then assayed for viability as above.

Flow Cytometry

All antibody staining was performed in HBSS buffer (Corning #21021CV) containing Pen/Strep (100 Units/mL; Fisher Scientific #MT30002CI), HEPES (10uM; Life Technologies #15630080) and SerumPlus II Medium Supplement (2%; Sigma #14009C). Bone marrow cells isolated from tibias, femurs, and iliac crests were combined for calculating total BM from each mouse. Peripheral blood, bone marrow, and spleen cells were suspended in complete HBSS ($1.0x10^8$ cells/mL) and incubated on ice for >20-minutes with desired antibodies. For analysis of human cells in PDX experiments – anti-human CD45-APC (BioLegend #368512), anti-human CD7-FITC (BioLegend #343104). For murine transplant experiments, as needed - CD45.2-BV421 (BioLegend #109831), CD45.1-FITC (BioLegend #110706), B220-APCcy7 (BioLegend

#103224), Gr-1-APCcy7 (BioLegend #108424), Mac-1-APCcy7 (BioLegend #101226), CD3e-APCcy7 (BioLegend #100330), Ter119-APCcy7 (BioLegend #116223), CD48-PECy7 (BioLegend #103424), CD150-PE (BioLegend #115904), c-Kit-BV605 (BioLegend #105847), Ly-6A/E (Sca-1)-APC (BioLegend # 122512). Dead cells were excluded with 7AAD (BioLegend #420404). Cell sorting was performed using MoFlo (Beckman Coulter) and FACS Aria II (BD) flow cytometers. Flow cytometric cell analysis was performed using Attune NxT (ThermoFisher Scientific) and FACS Aria II (BD) flow cytometers. Acquired flow cytometry data were analyzed with FlowJo software (Tree Star).

CRISPR Nucleofection and VAF determination

Synthetic guide RNAs (gRNAs) were designed using the UCSC Genome browser software. gRNAs were designed to target functional domains of *DNMT3A* and *BIRC5* to maximize specificity (lowest off-target effects) and efficiency (on-target cleavage efficiency). The four highest scoring gRNAs were tested by nucleofecting HEL and Jurkat cells. DNA was extracted from cells and sent for next generation sequencing (NGS) 48-hours post-nucleofection. gRNAs with the highest targeting efficiency were selected for experimentation. For both primary human T-ALL cells and T-ALL cell lines, nucleofection was performed using Cas9/ribonucleoprotein (IDT #1074181) complexed with gRNAs using the Neon system (Thermo Fisher Scientific). Nucleofection was carried out at 1600V with 3 pulses at a 10-millisecond width. Sequences of the gRNAs (Synthego) used for experimentation are as follows: *DNMT3A*_1012: UCCCCAGCAUCGGACCCCAC; *DNMT3A*_1014: CAGGCGUGGUAGCCACAGUG; *BIRC5*: ACUUACAUGGGGUCGUCAUC; *AAVS1*: GGGGCCACUAGGGACAGGAU (negative control). After nucleofection, primary human T-ALL cells were left to recover in StemSpan complete media described above. 48-hours post nucleofection, approximately 100,000 cells were collected, and genomic DNA was extracted to measure initial targeted variant allele fraction (VAF) using PCR amplicon-based deep sequencing. The following primers were used to generate amplicons:

AAVS1-forward: CACTCTTTCCCTACACGACGCTCTTCCGATCTACAGGAGGTGGGGGTTAGAC with *AAVS1*-reverse: GTGACTGGAGTTCAGACGTGTGCTCTTCCGATCTCCCCTATGTCCACTTCAGGA; *DNMT3A*_1012-forward: CACTCTTTCCCTACACGACGCTCTTCCGATCTCACCTCGTACTCTGGCTCGT with *DNMT3A*_1012-reverse: GTGACTGGAGTTCAGACGTGTGCTCTTCCGATCTCAGGAATGAATGCTGTGGAA; *DNMT3A*_1014-forward: CACTCTTTCCCTACACGACGCTCTTCCGATCTCACCTCGTACTCTGGCTCGT with *DNMT3A*_1014-reverse: GTGACTGGAGTTCAGACGTGTGCTCTTCCGATCTCAGGAATGAATGCTGTGGAA; *BIRC5*-forward: CACTCTTTCCCTACACGACGCTCTTCCGATCTCACTGAGAACGAGCCAGACTT with *BIRC5*-reverse: GTGACTGGAGTTCAGACGTGTGCTCTTCCGATCTGTTTAAGAAGCAATGAGGGTGG.

Gel purification of PCR amplicons was carried out using the Zymoclean Gel DNA Recovery kit (Zymo Research #D4008). Deep sequencing of libraries was carried out using the Illumina MiSeq platform and data was analyzed using CRISPResso2.

NSG mice were transplanted via tail vein injections with each mouse receiving approximately 250,000 nucleofected primary T-ALL cells. Bleeds were done 6-8 weeks post-transplant to quantify leukemia burden with purification of the hCD45+ hCD7+ cell fraction using flow cytometry to generate DNA for VAF analysis. Moribund mice were sacrificed, and bone marrow cells isolated from a combination of tibias, femurs, and iliac crests were used for genomic DNA extraction to measure targeted VAF at sacrifice using PCR amplicon-based deep sequencing. Bone marrow cells collected at sacrifice from primary transplants and 50,000 were transferred to secondary transplants.

Western Blot

Cells were washed twice with PBS, resuspended in 1X RIPA lysis buffer (EMD Millipore #20188) containing 1X of protease and phosphatase inhibitor (ThermoScientific #1861280) and sonicated for five minutes. The cell lysates were loaded on pre-made SDS-PAGE gels (Mini-PROTEAN TGX, BIO-RAD), transferred to polyvinylidene fluoride membranes (Fisher Scientific #IPVH00010), and blocked with 5% non-fat milk in Tris-buffered saline with 0.1% Tween 20 detergent (TBST). The blots were incubated with the indicated primary antibodies as required at 4°C overnight on a rocker. Blots were washed with TBST the following day and incubated with either mouse or rabbit secondary antibody conjugated with horseradish peroxidase (Fisher Scientific #55965-84-9 and #10794347) at room temperature on an orbital shaker for one hour. Detection was performed using chemiluminescence HRP substrate (Millipore #WBKLS0100). The blots were visualized on a Bio-Rad ChemiDOC Touch Imaging System (Bio-Rad Laboratories). The antibodies used were: DNMT3A (Santa Cruz Biotechnology #sc-365769), β-actin (Santa Cruz Biotechnology #sc-4778), Vinculin (Santa Cruz Biotechnology #sc-73614), Survivin (Cell Signaling Technology #2808S), phospho-STAT3 (Cell Signaling Technology #9145S) and phospho-STAT5 (Cell Signaling Technology #4322S). DNMT3A, β-actin, and Vinculin antibodies were used at concentration of 0.2 µg/mL. Survivin antibody was used at a concentration of 0.3 µg/mL.

T-ALL Cell Lines

The following cell lines were cultured in respective media: Jurkat (human T-ALL) - Gibco RPMI 1640 (Invitrogen #11875085) + 10% heat inactivated fetal bovine serum (HI-FBS); CUTTL1 (human T-ALL) - Gibco RPMI 1640 + 20% HI-FBS; MOHITO (mouse CD4+CD8+ double positive T-cell) – Gibco RPMI 1640 ATCC modification (Invitrogen # A1049101) + 20% HI-FBS + 10 ng/mL mouse IL-7 (Miltenyi #130-094-066) + 5 ng/mL mouse IL-2 (Miltenyi #130-094-054).

MOHITO cells were kindly provided by Dr. Ross Levine (Memorial Sloan Kettering). *DNMT3A* gene knockout in Jurkat and CUTTL1 cell lines were carried out as previously described using the CRISPR/Cas9 system together with gRNAs targeting *DNMT3A*. Following nucleofection, cells were left to recover in a 5% CO_2 incubator at 37ºC. 48-hours post nucleofection, cells were single cell sorted into 96 well cell culture plates. Sorted cells were left to recover in a 5% CO_2 incubator at 37ºC until cell colonies were visible. Single cell colonies were transferred to 6 well cell culture plates and cells were collected for genomic DNA extraction and western blot to confirm gene knockout.

siRNA

Validated siRNAs were used for transient gene inhibition in primary T-ALL patient cells. Multiple Silencer Select siRNAs were tested with the following being selected for final experimentation: *BIRC5* (ThermoFisher Scientific # s1457), Silencer Select Negative Control No. 2 siRNA (ThermoFisher Scientific #4390846). siRNAs were nucleofected into primary human T-ALL cells using the Neon system as described for CRISPR/Cas9. Nucleofected cells were incubated in a 5% CO_2 incubator at 37ºC for 48-hours. Following this, cell viability was determined using AnnexinV flow cytometry as described above.

RNA sequencing gene expression analysis (RNA-Seq)

Primary human T-ALL cells were treated with either dexamethasone (DEX), ruxolitinib (RUX), or the combination (DEX+RUX) for 24-hours in a 5% CO2 incubator at 37ºC. Both drugs were used at a final concentration of 10µM. Viable cells were isolated by flow cytometry and total RNA was extracted using the RNeasy Plus Mini kit (Qiagen #74136). The quality of the extracted RNA was assessed using a Bioanalyzer (Agilent Technologies). Samples with a RIN value of >8 were submitted for RNA sequencing. The SMARTer Ultra Low RNA kit (Clontech) was used to prepare libraries for RNA-seq. Sequencing was performed on an Illumina NovaSeq S4 2x150.

For analysis, Partek Flow Genomic Analysis software was used. Raw RNA-seq reads were aligned to the Ensembl release 105 (Human) using STAR version 2.7.3a. Alignment efficacy and read quality was assessed using quality control steps. Gene counts were normalized, and Gene Specific Analysis (GSA) was performed on the normalized counts to generate the differential analysis. Gene set enrichment analysis (GSEA) was performed to identify enriched molecular pathways and the associated gene sets. Primary data is available at dbGAP under accession phs003623.

Whole Genome Bilsufite Sequencing (WGBS)

DNA was extracted from T-ALL patient cells using the PureLink Genomic DNA Mini Kit (Thermo Fisher Scientific #K1820-02) and quantified using Qubit. 200ng of genomic DNA, including 0.2% Lambda DNA (N6-methyladenine-free; NEB) was fragmented in a final volume of 50μL using the Covaris LE220 targeting ~350bp inserts. A 1.5x AMPure clean-up was performed post fragmentation resulting in a final volume of 20 μL. Fragmented DNA was bisulfite converted with the EZ-96 DNA Methylation-Gold Mag Prep Kit (Zymo Research) according to manufacturer recommendations. Whole genome bisulfite (WGBS) libraries were constructed with ~100ng of bsDNA using the xGen Methyl-Seq Library Prep Kit (IDT) and unique dual indexes (IDT). Nine PCR cycles were performed during the indexing PCR step, followed by a final 0.85x AMPure cleanup. Final libraries were assessed on the bioanalyzer instrument for average library size and concentration measured by qPCR using KAPA library quantification kits (Roche). Libraries were sequenced using 2x150 paired end reads on an Illumina NovaSeq X Plus.

The average sequencing coverage depth was 21.2x (range 15.3x - 26.4x). Paired end sequencing reads were trimmed to remove adapters and low-quality sequence using TrimGalore (v0.4.4_dev, --paired --three_prime_clip_R1 10 --clip_R2 10). Trimmed reads were then mapped to the human genome (hg38) using bismark (v0.18.2, default parameters) and converted to per

CpG methylation data using the included bismark_extractor. Bismark output files were filtered to only include CpGs with coverage >5 in every sample and limited to autosomal CpGs only. The number of sites was then filtered to the 10,000 CpGs with the highest variance in mCG/CG across all samples (using the var() function in R). Samples were clustered using Euclidean distance and complete linkage, and cluster heatmaps were plotted using pheatmap (scale=”none”). In R the data were centered using the scale() function and principle components computed using prcomp() (scale.= TRUE). Scree plots were visualized with the fviz_eig() function in the factoextra R package. 2D PCA plots were generated with the autoplot() function in the ggfortify R package and 3D PCA were generated using the plot3d() function in the rgl R package. DMRs were computed using DSS (v2.48.0) between *DNMT3A* wildtype and mutant samples. The test function DMLtest was run with smoothing=TRUE and otherwise default parameters. DMRs were then computed with a *p*-value threshold of 0.01. DMRs have a minimum length of 50 bp, must contain 3 or more CpGs, and DMRs within 50 bp are merged (DSS default parameters). DMRs were filtered after calling based on the absolute value of the mean methylation difference (>= 0.3) and limited to autosomal DMRs only. DMRs were annotated using Homer (default settings) and the Gencode v45 basic human gene annotations. DMR clustering was performed by first calculating the average methylation (mCG/CG) across the DMR using bedtools map across all samples. DMRs missing methylation data in one or more samples were removed. Samples were clustered using Euclidean distance and complete linkage, and cluster heatmaps were plotted using pheatmap (scale=”none”). For genome browser tracks, bigWig files were created using bedGraphToBigWig from the UCSC Genome Browser toolkit. Primary data is available at dbGAP under accession phs003623.

Statistics

Student t-test, one-way, and two-way ANOVA were used for statistical comparisons where appropriate. Survival curves were analyzed using a Mantel-Cox logrank test. Significance is indicated using the following convention: *p<0.05, **p<0.01, ***p<0.001,****p<0.0001. All graphs represent mean ± S.E.M.

SUPPLEMENTAL REFERENCES

1. Kaneda M, Okano M, Hata K, et al. Essential role for de novo DNA methyltransferase Dnmt3a in paternal and maternal imprinting. *Nature*. 2004;429(6994):900-903.

2. Stanger BZ, Datar R, Murtaugh LC, Melton DA. Direct regulation of intestinal fate by Notch. *Proc Natl Acad Sci U S A*. 2005;102(35):12443-12448.

3. Pear WS, Aster JC, Scott ML, et al. Exclusive development of T cell neoplasms in mice transplanted with bone marrow expressing activated Notch alleles. *J Exp Med*. 1996;183(5):2283-2291.

4. Aries IM, Bodaar K, Karim SA, et al. PRC2 loss induces chemoresistance by repressing apoptosis in T cell acute lymphoblastic leukemia. *J Exp Med*. 2018;215(12):3094-3114.
